# Relationship Between Amino Acid Intake in Maternal Diet and Risk of Gestational Diabetes Mellitus: Results from the BORN 2020 Pregnant Cohort in Northern Greece

**DOI:** 10.3390/nu17010173

**Published:** 2025-01-02

**Authors:** Antigoni Tranidou, Antonios Siargkas, Emmanuela Magriplis, Ioannis Tsakiridis, Aikaterini Apostolopoulou, Michail Chourdakis, Themistoklis Dagklis

**Affiliations:** 1 3rd Department of Obstetrics and Gynecology, School of Medicine, Faculty of Health Sciences, Aristotle University of Thessaloniki, 541 24 Thessaloniki, Greece; antigoni.tranidou@gmail.com (A.T.); antonis.siargkas@gmail.com (A.S.); igtsakir@auth.gr (I.T.); katapost@yahoo.gr (A.A.); 2 Laboratory of Hygiene, Social & Preventive Medicine and Medical Statistics, School of Medicine, Faculty of Health Sciences, Aristotle University of Thessaloniki, 541 24 Thessaloniki, Greece; mhourd@auth.gr; 3 Department of Food Science and Human Nutrition, Agricultural University of Athens, Iera Oos 75, 118 55 Athens, Greece; emagriplis@eatsmart.gr

**Keywords:** maternal nutrition, dietary protein, amino acids, biological value, GDM risk

## Abstract

**Background/Objectives:** Maternal amino acid intake and its biological value may influence glucose regulation and insulin sensitivity, impacting the risk of developing gestational diabetes mellitus (GDM). This study aimed to evaluate the association between amino acid intake from maternal diet before and during pregnancy and the risk of GDM. **Methods:** This study is part of the ongoing BORN2020 epidemiological Greek cohort. A validated semi-quantitative Food Frequency Questionnaire (FFQ) was used. Amino acid intakes were quantified from the FFQ responses. A multinomial logistic regression model, with adjustments made for maternal characteristics, lifestyle habits, and pregnancy-specific factors, was used. **Results:** A total of 797 pregnant women were recruited, of which 14.7% developed GDM. Higher cysteine intake during pregnancy was associated with an increase in GDM risk (adjusted odds ratio [aOR]: 5.75; 95% confidence interval [CI]: 1.42–23.46), corresponding to a 476% increase in risk. Additionally, higher intakes of aspartic acid (aOR: 1.32; 95% CI: 1.05–1.66), isoleucine (aOR: 1.48; 95% CI: 1.03–2.14), phenylalanine (aOR: 1.6; 95% CI: 1.04–2.45), and threonine (aOR: 1.56; 95% CI: 1.0–2.43) during pregnancy were also associated with increased GDM risk. Furthermore, total essential amino acid (EAA) (aOR: 1.04; 95% CI: 1.0–1.09) and non-essential amino acid (NEAA) (aOR: 1.05; 95% CI: 1.0–1.1) intakes during pregnancy were also linked to an increased risk of GDM. A secondary dose–response analysis affected by timing of assessment revealed that higher intake levels of specific amino acids showed a more pronounced risk. **Conclusions:** Optimizing the balance of certain amino acids during pregnancy may guide personalized nutritional interventions to mitigate GDM risk.

## 1. Introduction

Due to its rising prevalence, recent research has shifted focus to addressing modifiable risk factors for reducing the risk of developing gestational diabetes mellitus (GDM) [1,2]. Among these, maternal nutritional status has emerged as a critical area of focus, considering its potential to influence metabolic health and promote a healthy pregnancy course, thereby lowering GDM incidence [3,4]. Published data have investigated the role of dietary proteins either before conception and during pregnancy, with results indicating that source (animal or vegetable) and protein quality may have a differential influence on GDM risk [5,6]. Amino acids (AAs) are the fundamental building blocks of proteins, and they reflect the nutritional value of dietary sources [7]. Historically, AAs have been identified as essential amino acids (EAAs) and non-essential amino acids (NEAAs) [8]. EAAs, including histidine, isoleucine, leucine, lysine, methionine, phenylalanine, threonine, tryptophan, and valine, cannot be synthesized by the human body, and thus they need to be obtained through diet. Their role is to support optimal health and provide energy for growth requirements [9]. On the other hand, NEAAs (alanine, asparagine, aspartic acid, glutamic acid, and serine) are synthesized endogenously under normal conditions [10]. However, emerging evidence suggests that they may also serve as dietary essentials for achieving full genetic potential growth, development, glucose and lipid metabolism, and endocrine status, among others, proposing a paradigm shift in protein nutrition [11]. Additionally, some AAs, such us arginine and cysteine, are classified as conditionally essential, as their endogenous synthesis may become insufficient during physiological stress or illness [12].

Few studies have focused on the association between specific AA subgroup profiles during pregnancy and GDM risk; branch chain amino acids (BCAAs), a subgroup of EAAs including leucine, isoleucine, and valine, have been implicated in metabolic disorders, like increased insulin resistance and type 2 diabetes [13]. Elevated circulating levels of BCAAs have been observed in obese, non-pregnant individuals, likely due to impaired BCAA catabolism in adipose tissue. This leads to their accumulation in the bloodstream, contributing to insulin resistance and metabolic dysregulation, especially when BCAAs are consumed through dietary sources [9,14]. Similarly, aromatic amino acids (AAAs) including phenylalanine, tyrosine, and tryptophan, are also involved in pathways affecting oxidative stress and homeostasis. In a study conducted in pregnant women, elevated circulating levels of AAAs, particularly phenylalanine and tryptophan, were associated with an increased risk of GDM, with significant interactions noted between these AAs and gut microbiota-related metabolites [15]. These findings were based on blood-based measurements of AAAs, which reflect endogenous metabolic activity rather than dietary intake. The study did not explicitly differentiate whether these amino acids were derived from food, supplements, or internal metabolic processes, focusing instead on their circulating levels and their role in disrupting metabolic balance. This points to the potential of AAAs as biomarkers for GDM risk and highlights the need to better understand how dietary intake contributes to their circulating levels. Moreover, AAAs, through their influence on tryptophan metabolism and serotonin synthesis, have been linked to oxidative stress, which can further exacerbate insulin resistance [16]. A study by Gao et al. (2022) found that while fasting levels of BCAAs and AAAs did not differ significantly between women with and without GDM, a delayed and blunted decrease in these amino acids was observed after glucose ingestion in the GDM group. This delayed metabolic response suggests the underlying dysregulation of amino acid metabolism in GDM, which may be a marker of impaired insulin sensitivity [17]. In addition, BCAA and AAAs have been implicated in the development of insulin resistance and type 2 diabetes (T2D). Elevated circulating levels of these amino acids are observed not only in individuals with obesity, a known risk factor for T2D, but also in individuals with prediabetes and diabetes. A meta-analysis has confirmed that increased levels of BCAAs, particularly leucine and isoleucine, correlate with an increased risk of T2D, as well as with the worsening of insulin resistance over time. Similar associations have been observed with AAAs [18].

Existing research stresses the importance of dietary protein quality and source in relation to GDM risk [6,19]. However, due to controversial results and low evidence, there is a need for further studies focusing on individual AA intake and their influence on GDM development [20], using a prospective design, where temporality can be at least established. Additionally, dietary habits may vary significantly across countries, and the impact of these differences on GDM risk needs further clarification.

This study aimed to address this gap by examining the association between AA intake from maternal diets before and during pregnancy and the risk of GDM in a Greek pregnant cohort. Based on prior research suggesting a role for amino acids in metabolic regulation, we hypothesized that higher maternal AA intake, obtained through diet before and during pregnancy, may increase GDM risk.

## 2. Materials and Methods

### 2.1. Study Design and Participants

This study is a component of the BORN2020 study, a large scale longitudinal prospective cohort study of pregnant women, designed to collect and analyze the nutritional and physical data of pregnant women in Northern Greece [21]. All pregnant women were recruited at the 3rd Department of Obstetrics and Gynecology, School of Medicine, Faculty of Health Sciences Aristotle University of Thessaloniki, Greece. The selection criteria used to determine eligibility were (i) ongoing pregnancy, (ii) age > 18 years old, and (iii) very good understanding of the Greek language. The exclusion criteria included (i) women on any specific nutritional program due to any health issue (e.g., malabsorption syndrome, inflammatory bowel disease, etc.) and (ii) a diagnosis of certain pre-existing medical conditions, including diabetes mellitus (type 1 or type 2), chronic hypertension, and autoimmune diseases.

### 2.2. Dietary Assessment

Participating women were orally interviewed by trained personnel in two different time settings, one covering dietary intake for up to six months before conception (period A) and the other (period B) covering the period up until mid-gestation, right before the oral glucose tolerance test (OGTT). A validated semi-quantitative Food Frequency Questionnaire (FFQ) was used to collect the data [22], and it required about 20 min for completion. The first interview was performed during the 1st trimester routine prenatal visit (11^+0^ to 13^+6^ weeks of gestation), while the second interview was performed at 21–23 weeks of gestation, before routine GDM screening.

Nutritional data were entered into Nutrisurvey, a software tool for dietary assessment. Based on the participants’ reported intake, AA intake was estimated by taking the sum of the nutritional analysis of all foods consumed for each FFQ question.

The AA categories included EEAs (histidine, isoleucine, leucine, lysine, methionine, phenylalanine, threonine, tryptophan, and valine), NEAAs (alanine, aspartic acid, glutamic acid, and serine), and certain Aas, which were categorized into BCAAs (leucine, isoleucine, and valine) and AAAs (phenylalanine, tyrosine, and tryptophan) based on their structure.

### 2.3. Diagnosis of GDM

GDM was diagnosed following the guidelines established by the Hellenic Society of Obstetricians and Gynecologists. According to these, all pregnant women are routinely screened for GDM between 24 and 28 weeks of gestation, based on the findings of the HAPO study [23]. The screening involves a 75 g oral glucose tolerance test (OGTT), where glucose levels are measured at fasting (<92 mg/dL), 60 min after ingestion (<180 mg/dL), and 120 min after ingestion (<153 mg/dL). A diagnosis of GDM is made if one or more of these values meet or exceed the established thresholds. In our study, we recruited women who attended the antenatal clinic for their ultrasound screening at 11^+0^–13^+6^ weeks of gestation. It is important to note that women who underwent early screening for GDM before 24 weeks (typically conducted at 7–9 weeks of gestation for those with risk factors) were excluded from our analysis. The diagnosis of GDM was based on the detection of abnormal glucose values during the routine OGTT conducted between 24 and 28 weeks.

### 2.4. Statistical Analysis

For continuous variables, normality was assessed with the Shapiro–Wilk test. Variables with a normal distribution were expressed as means with standard deviations (SDs), while non-normally distributed variables were expressed as medians with interquartile ranges (IQRs). Categorical variables were presented as frequencies and percentages, and comparisons between groups were performed using the Chi-square of Fisher’s exact test. The R language (v4.2.1.) was used for statistical analysis. Comparisons between the groups were conducted based on the normality of variables (*t*-tests and the Mann–Whitney U test).

The relationship between AA intake and GDM risk was analyzed using a multinomial logistic regression model to estimate adjusted odds ratios (aORs) with 95% confidence intervals (CIs). The model was adjusted for potential confounders, including maternal age, physical activity, energy intake, BMI, gestational weight gain up to 24 weeks of gestation, supplementation use (binary variable), smoking, thyroid conditions (such as hypothyroidism, Hashimoto’s thyroiditis, and hyperthyroidism), use of assisted reproductive technologies (ART), and parity. A dose–response analysis using a quartile approach was applied to analyze amino acid intake in relation to GDM risk, using multinomial logistic regression and adjusting for the same covariates.

Additionally, the biological value of dietary protein sources was evaluated by calculating the percentage of EEAs relative to the total AA intake [24], using data obtained from Nutrisurvey.

## 3. Results

The differences observed in maternal characteristics between the groups (GDM vs. non-GDM) are presented in Table 1. Moreover, as seen in Table 2, among EAAs, no significant relationships were observed for individual AAs before pregnancy and GDM risk. The aORs ranged from 0.8 (95% CI: 0.20–3.00) for tryptophan to 0.97 (95% CI: 0.8–1.2) for lysine. Similarly, for NEAAs, there were no significant findings. The aOR for alanine was 0.9 (95% CI: 0.6–1.2), and the aOR for glutamic acid was 0.94 (95% CI: 0.9–1.03). BCAAs and AAAs both collectively lacked statistical significance with GDM risk [aOR of 0.97 (95% CI: 0.90–1.1); aOR of 0.9 (95% CI: 0.8–1.1)].

When examining the %EEAs in total AA intake, no significant relationship was observed (aOR: 1.10; 95% CI: 0.95–1.3). In addition, total EAA intake (aOR: 0.99; 95% CI: 0.96–1.03) and total NEAA intake (aOR: 0.99; 95% CI: 0.95–1.02) showed no significant relationships with GDM. Overall, no pre-pregnancy AA intake, including EAAs and NEAAs, demonstrated a statistically significant relationship with GDM risk in our study cohort.

Table 3 shows the relationship between higher intake of AA during pregnancy and GDM risk. Total EAA (aOR: 1.04, 95% CI: 1.00–1.1) and total NEAA (aOR: 1.05, 95% CI: 1.00–1.1) intakes were significantly associated with GDM risk. Among specific AAs, significant relationships were observed (i) for the EAA group for isoleucine (aOR: 1.5; 95% CI: 1.03–2.1), phenylalanine (aOR: 1.60; 95% CI: 1.04–2.5) and threonine (aOR: 1.6; 95% CI: 1.00–2.4) and (ii) for the NEAA group for cysteine (aOR: 5.8, 95% CI: 1.42–23.46) and aspartic acid (aOR: 1.3, 95% CI: 1.1–1.7).

### Tables

Table S1 shows the shift in dietary protein load in GDM vs. non-GDM individuals from pre-pregnancy to pregnancy. These data were previously reported in another analysis from the same study [25]. Protein intake increased significantly during pregnancy, as evidenced by a higher absolute protein intake (aOR: 1.0, 95% CI: 1.00–1.01). However, protein as a percentage of energy intake did not show a significant change (aOR: 1.02, 95% CI: 0.98–1.07). Both vegetable and animal protein intakes also increased significantly during pregnancy (aOR: 1.01, 95% CI: 0.99–1.03; aOR: 1.00, 95% CI: 1.00–1.01).

Table S2 shows the results of a post hoc power analysis conducted to evaluate the statistical power of the association between dietary amino acids and GDM risk. For Period A, no significant relationships between dietary amino acids and GDM risk were identified. For the statistically significant results in Period B, namely isoleucine (aOR: 1.48, 95% CI: 1.03–2.14, *p* = 0.034, power = 1), threonine (aOR: 1.56, 95% CI: 1.00–2.43, *p* = 0.048, power = 1), cysteine (aOR: 5.75, 95% CI: 1.42–23.46, *p* = 0.014, power = 1), aspartic acid (aOR: 1.32, 95% CI: 1.05–1.66, *p* = 0.016, power = 1), and arginine (aOR: 1.53, 95% CI: 1.11–2.10, *p* = 0.008, power = 1), the power analysis suggests strong confidence in the reliability of the relationships. On the other hand, for EAAs (aOR: 1.04, 95% CI: 1.00–1.09, *p* = 0.02, power = 0.161), and NEAAs (aOR: 1.05, 95% CI: 1.00–1.10, *p* = 0.026, power = 0.195), the relatively low power (0.161 and 0.195, respectively) indicates that these results should be interpreted with caution, and further studies with larger sample sizes are needed to confirm these findings.

Table S3 shows the results of a dose–response analysis by applying quartiles (Q4) for amino acid intakes and evaluating their association with the risk of GDM in Period A and Period B. For the statistically significant results in Period A, glycine (Q4 medium, *p* = 0.044 *, aOR = 1.87, 95% CI: 1.02–3.49, power = 1) was significantly associated with an increased risk of GDM. Other amino acids were not statistically significant.

In Period B, the dose–response analysis revealed several statistically significant associations with strong statistical power, suggesting robust findings. For instance, isoleucine (Q4 medium, *p* = 0.001 **, aOR = 2.97, 95% CI: 1.54–5.97, power = 1), isoleucine (Q4 high, *p* = 0.042 *, aOR = 2.15, 95% CI: 1.03–4.62, power = 1), and isoleucine (Q4 very_high, *p* < 0.001 ***, aOR = 4.2, 95% CI: 1.91–9.58, power = 1) demonstrated significant associations with a substantially increased risk of GDM. Similarly, leucine (Q4 medium, *p* = 0.015 *, aOR = 2.25, 95% CI: 1.18–4.42, power = 1), leucine (Q4 high, *p* = 0.01 *, aOR = 2.47, 95% CI: 1.25–5.02, power = 1), and leucine (Q4 very_high, *p* = 0.009 **, aOR = 2.89, 95% CI: 1.32–6.49, power = 1) exhibited strong positive associations. Methionine (Q4 medium, *p* = 0.016 *, aOR = 2.2, 95% CI: 1.17–4.28, power = 1), methionine (Q4 very_high, *p* = 0.005 **, aOR = 2.95, 95% CI: 1.38–6.44, power = 1), and phenylalanine (Q4 medium, *p* = 0.009 **, aOR = 2.37, 95% CI: 1.25–4.61, power = 1) also demonstrated significant associations with increased GDM risk. Additionally, threonine (Q4 medium, *p* = 0.007 **, aOR = 2.39, 95% CI: 1.28–4.62, power = 1), threonine (Q4 very_high, *p* = 0.009 **, aOR = 2.8, 95% CI: 1.31–6.16, power = 1), tryptophane (Q4 medium, *p* = 0.04 *, aOR = 1.96, 95% CI: 1.04–3.79, power = 1), and tryptophane (Q4 very_high, *p* = 0.029 *, aOR = 2.35, 95% CI: 1.09–5.16, power = 1) were significantly associated with GDM risk. Other amino acids such as arginine (Q4 very_high, *p* = 0.024 *, aOR = 2.26, 95% CI: 1.11–4.64, power = 1), tyrosine (Q4 high, *p* = 0.023 *, aOR = 2.13, 95% CI: 1.12–4.16, power = 1), EAAs (Q4 very_high, *p* = 0.012 *, aOR = 2.68, 95% CI: 1.25–5.85, power = 1), BCAAs (Q4 medium, *p* = 0.002 **, aOR = 2.87, 95% CI: 1.49–5.76, power = 1), BCAAs (Q4 high, *p* = 0.007 **, aOR = 2.7, 95% CI: 1.34–5.65, power = 1), and BCAAs (Q4 very_high, *p* = 0.005 **, aOR = 3.22, 95% CI: 1.43–7.46, power = 1) also showed strong associations with GDM risk. Finally, AAAs (Q4 medium, *p* = 0.023 *, aOR = 2.12, 95% CI: 1.12–4.12, power = 1), AAAs (Q4 high, *p* = 0.04 *, aOR = 2.06, 95% CI: 1.04–4.16, power = 1), and AAAs (Q4 very_high, *p* = 0.017 *, aOR = 2.59, 95% CI: 1.19–5.76, power = 1) were also significantly associated with increased risk of GDM.

Table S4 presents the quartile cut points used for the dose–response analysis. Table S5 reports the aORs and significance levels for all adjusted variables included in the regression models.

## 4. Discussion

### 4.1. Main Findings

After adjusting for multiple significant confounders, this study’s main findings were as follows: (i) the intake of AAs before pregnancy is not associated with GDM; (ii) during pregnancy, the intake of AAs was positively associated with GDM both for EAAs and NEAAs; and (iii) higher dietary intakes of isoleucine, phenylalanine, threonine, cysteine, aspartic acid, and arginine during pregnancy were significantly associated with elevated GDM risk.

### 4.2. Secondary Dose–Response Analysis Using Quartiles

In addition to the main findings, a secondary analysis was conducted to explore the dose–response relationship between amino acid intake during pregnancy and GDM risk by categorizing intake into quartiles. This analysis aimed to investigate whether higher levels of specific amino acids, particularly in the upper quartiles, were more strongly associated with GDM risk.

The results from the quartile analysis showed the following key findings: (i) during Period A (pre-pregnancy), intakes of glycine in the medium quartile and intakes of EAAs in the highest quartile increased GDM risk; (ii) during Period B (pregnancy), isoleucine, leucine, valine, lysine, methionine, phenylalanine, threonine, tryptophan, aspartic acid, cysteine, proline, arginine, tyrosine, EAAs, NEAAs, BCAAs and AAs in the upper quartiles were significantly associated with an increased risk of GDM during pregnancy, suggesting a potential dose–response relationship between amino acid intake and GDM risk.

The results from the quartile analysis further support the findings from the main analysis, where EAA and NEAA intake during pregnancy were positively associated with GDM risk. However, the secondary analysis provides additional clarity by demonstrating that the relationship between amino acid intake and GDM risk is dose-dependent, with higher intake levels in the upper quartiles showing stronger associations with GDM risk. This dose–response relationship emphasizes the critical role of higher levels of intake rather than just the presence of specific amino acids.

### 4.3. Interpretation of the Findings

As previously mentioned, no significant relationships were observed between pre-pregnancy AA intake and GDM risk in our cohort. This suggests that maternal metabolic adaptations during pregnancy might modulate AA metabolism, making mid-gestation a crucial window for evaluating the impact of dietary AAs on GDM risk. This observation is supported by a recent meta-analysis, which found that total protein intake before pregnancy is not associated with GDM, while during pregnancy there is a positive correlation [26]. This could be attributed to the increase in basal endogenous glucose production in healthy pregnant women after mid-gestation, primarily due to increased hepatic output and the decrease in maternal insulin sensitivity during pregnancy. If a pregnant woman’s pancreatic β-cells cannot adequately boost insulin secretion to compensate for this insulin resistance, GDM may develop [27]. As a result, maternal protein and thus AA intake during mid-gestation hold greater significance for GDM risk than pre-pregnancy intake.

Our study revealed a statistically significant relationship between higher dietary intake of certain AAs—namely isoleucine, phenylalanine, threonine, cysteine, aspartic acid, and arginine—and elevated GDM risk during pregnancy. The highest odds for GDM were observed for the increased intake of cysteine (aOR: 5.8). While the relationship is notable, the wide CI suggests potential limitations in the statistical power of our analysis, likely due to sample size or variability in intake levels. Nevertheless, the relationship of cysteine with GDM risk in this study warrants further investigation, as no prior studies have directly examined this AA. However, cysteine’s role in glutathione synthesis and oxidative stress pathways might explain its strong association with GDM risk. Elevated oxidative stress may impair insulin receptor signaling and contribute to the metabolic dysregulation observed in GDM pregnancies [28].

Among the BCAAs, isoleucine was the one with the highest odds for GDM (aOR: 1.5) and reached statistical significance, whereas the other BCAAs showed a positive but not statistically significant relationship. These results are consistent with prior research, which has shown that elevated levels of BCAAs, isoleucine, and valine, specifically, are predictive of GDM and type 2 diabetes mellitus due to their contribution to impaired insulin signaling and pancreatic β-cell dysfunction [29,30,31,32]. Isoleucine appears to be systematically associated with a higher risk of GDM compared to other BCAAs. Yang et al. identified only isoleucine as statistically significant among the BCAAs in relation to GDM, a finding similar to our results [31]. Conversely, leucine was not associated with GDM when adjusted for confounders [29,31], and findings regarding valine were inconsistent; while most studies reported increased valine levels in the GDM population, these associations did not consistently reach statistical significance [31]. In the general population, BCAAs have been connected to insulin resistance and metabolic dysfunction in both pediatric and adult populations, including pregnant and non-pregnant groups [33]. These amino acids have been shown to negatively influence glucose regulation [34]. Elevated BCAA levels have been associated with obesity and insulin-resistant conditions due to disruptions in protein anabolism and impaired insulin signaling [33,35,36,37].

It is noteworthy that the cumulative intake of BCAAs during pregnancy was not associated with GDM; thus, we validate the similar results of a nested matched case control devised by Yang et al. [31]. In this way, our study demonstrated that not all BCAAs equally influence insulin resistance during pregnancy, with isoleucine emerging as a predominant factor. Elevated isoleucine levels may more effectively activate the PI3K/AKT/mTORC1 signaling pathway compared to other BCAAs. This pathway regulates insulin signaling and metabolic processes such as protein synthesis and cell growth [33]. Chronic mTORC1 activation can disrupt insulin signaling by hyperactivating S6K1, which phosphorylates insulin receptor substrates on serine residues, impairing insulin signal transmission and promoting insulin resistance [33]. During pregnancy, this exacerbates the natural decrease in insulin sensitivity, potentially leading to GDM [27]. In our study, all AAAs exhibited increased aOR for GDM, with phenylalanine showing a statistically significant relationship (aOR: 1.6). This finding is consistent with previous research that consistently links phenylalanine to GDM. Tyrosine was significantly associated with GDM in one of two prior studies that adjusted for confounders [29,31], while tryptophan showed no significant relationship in either study [29,31]. Although the cumulative intake of AAAs did not reach statistical significance in our analysis, it had the highest adjusted odds ratio among amino acid groups, suggesting a trend toward increased GDM risk. Similarly, a nested matched case–control study of 2802 pregnant women found that AAAs were the only amino acid group significantly associated with GDM [31]. These results indicate that specific AAAs, particularly phenylalanine and tyrosine, may primarily drive the relationship between AAAs and insulin resistance or GDM risk, rather than the entire group. In our study, tyrosine did not remain significant after adjusting for confounders, and previous studies have shown inconsistent results regarding its role [29,31]. Although tyrosine was associated with a higher odds ratio for GDM, larger population studies are needed to confirm its significance and clarify its role. A prospective cohort study of 2422 normoglycemic individuals followed for 12 years found that both phenylalanine and tyrosine were linked to an increased risk of developing type 2 diabetes, potentially due to impaired insulin signaling and pancreatic β-cell dysfunction [32]. Elevated intake of AAAs has been associated with impaired insulin signaling and reduced glucose clearance, contributing to insulin resistance and a higher risk of GDM [38]. Tyrosine, in particular, can be converted into dopamine, which inhibits insulin secretion and further impairs glycemic control [39]. In addition, AAAs and their derivatives can induce oxidative stress and inflammation, damaging pancreatic β-cells and exacerbating insulin resistance [40,41]. No further pathophysiologic mechanisms have been investigated for the specific AAAs.

Similarly, aspartic acid and threonine (glucogenic AAs) were also significantly associated with GDM. The findings for aspartic acid are in accordance with relevant studies [29,31]. Glutamate and aspartic acid are involved in *N*-methyl-D-aspartate (NMDA) activation; the inhibition of NMDA increases glucose-stimulated insulin secretion, improving glucose tolerance [28,42]. Threonine, an EAA, plays a crucial role in glucose metabolism by contributing to gluconeogenesis through its degradation to α-ketobutyric acid, a precursor for glucose synthesis [43]. While threonine is generally associated with maintaining glucose homeostasis, our findings suggest that elevated threonine levels may increase the risk of GDM. This could be attributed to specific metabolic shifts during pregnancy, including a shift toward increasing protein intake, which is the case for our population, or individual variations in AA metabolism that amplify threonine’s impact on glucose regulation.

In our study, during pregnancy, the total intake of EAAs and NEAAs was associated with a modest increase in GDM risk, while the percentage of EAAs was not statistically significant. This could be explained by the shift in dietary protein load, which may strain metabolic pathways involved in glucose regulation, potentially contributing to the development of GDM.

In addition, several factors have been reported to affect AA digestibility and overall protein quality [44]. These include but are not limited to food processing techniques, such as heating and alkaline treatments, which are widely used in the preparation and manufacturing of protein-based foods, including soy protein isolates, casein, and wheat protein. These methods, while necessary for improving food texture, flavor, and shelf life, may alter the structure of proteins, leading to the formation of compounds such as Maillard reaction products, oxidized sulfur AAs, D-amino acids, and lysinoalanine. In humans, these compounds are poorly digestible and reduce the bioavailability of EAAs like lysine, ultimately lowering the nutritional quality of protein sources. For example, Maillard reaction products formed during heating reduce lysine availability, while lysinoalanine, a derivative from alkaline processing, has been shown to exhibit nephrotoxic effects when consumed in large amounts. These effects are important for consideration, particularly for populations reliant on highly processed foods as their primary protein sources.

### 4.4. Direction for Future Research

Future research should focus on conducting larger prospective cohort studies with robust adjustments for significant confounders to better elucidate the relationship between specific amino acid intake and GDM risk. Additionally, intervention studies that specifically assess changes in the intake of individual amino acids, with GDM as the primary outcome, are crucial to determining whether dietary modifications can improve outcomes. Such studies would provide valuable insights into potential causal pathways and inform targeted dietary recommendations for GDM prevention and management.

### 4.5. Strengths and Limitations

This study assessed AA intake both before conception and antenatally, capturing dietary habits prior to disease onset and enhancing the ability to infer causality between macronutrient intake and GDM risk. A key strength of this study is the use of a validated FFQ specifically designed for the Greek population, which provided reliable estimates of amino acid intake. This robust tool minimized recall bias and ensured consistency in dietary assessments, supporting the validity of the observed associations between amino acid intake and GDM risk. Additionally, this study’s substantial sample size of 797 pregnant women, including 117 diagnosed with GDM, may have provided adequate statistical power to detect significant relationships. Assessing dietary intake at two distinct time points allowed us to evaluate changes in AA intake, offering useful insights into how these shifts relate to GDM risk. Moreover, for the statistically significant results, the high power values (all equal to 1) for most amino acids suggest that these associations are highly reliable. In particular, cysteine and arginine showed exceptionally strong associations with GDM risk (for the during pregnancy period), as indicated by large aORs (5.75 and 1.53, respectively), reinforcing the robustness of these findings. However, while EAAs and NEAAs were statistically significant, their relatively low power values (0.161 and 0.195) suggest caution in interpreting these results, as they may reflect weaker associations or be subject to sampling variability. Overall, the post hoc analysis demonstrated that with high power for most variables, the statistically significant associations in Period B were robust and reflected a meaningful relationship between amino acid intake and GDM risk. In addition, the dose–response analysis showed high power values (mostly equal to 1) in Period B, suggesting that these findings were robust, and the statistical power was sufficient to detect real relationships.

Regarding this study’s limitations, most of the observed effect sizes were small, which may limit both statistical power and clinical significance. The use of self-reported dietary data is subject to recall bias and inaccuracies, although efforts were made to minimize this by conducting interviews with experienced researchers. In addition, no interaction terms were tested in this study, as our primary focus was on the main effects of amino acids and other variables on the outcomes of interest. While the FFQ used in this study was not specifically validated for AA quantification, it was validated for major food groups that contain these AAs. Furthermore, the observational nature of this study precluded definitive conclusions about causality, as unmeasured confounders or reverse causation could not be fully ruled out. Lastly, the possibility of misclassification bias in dietary assessments may also have influenced the findings, as the precise categorization of dietary intake is challenging.

## 5. Conclusions

This study found that higher intakes of amino acids AAs during pregnancy, particularly essential amino acids EAAs and NEAAs, were positively associated with an increased risk of GDM. Specifically, the intakes of isoleucine, phenylalanine, threonine, cysteine, aspartic acid, and arginine were significantly linked to elevated GDM risk. The secondary dose–response analysis using quartiles further revealed that higher intake levels of isoleucine, leucine, methionine, phenylalanine, threonine, tryptophan, arginine, tyrosine, and EAAs were more strongly associated with GDM risk, supporting a dose-dependent relationship. These findings suggest that dietary interventions focusing on amino acid intake during pregnancy, especially those associated with insulin resistance, could help to manage GDM risk. However, given the complexity of amino acid metabolism and individual variability, further research is needed to clarify these relationships and inform practical clinical guidelines for managing GDM risk through dietary modifications.

## Figures and Tables

**Table 1:** Characteristics of women with GDM diagnosis and women without GDM diagnosis.

Maternal Variables	GDM (*N* = 117)	Non-GDM (*N* = 680)	*p* Value	Comparison
MA (mean, SD)	34.2 (±4.5)	32.1 (±4.9)	*p* < 0.0001 ***	↑ in GDM
MA > 35 (*n*%)	51 (43.6%)	186 (27.4%)	*p* < 0.001 ***	↑ in GDM
Weight before pregnancy (median (IQR))	65 (59, 79)	63 (57, 73)	0.008 **	↑ in GDM
Height (centimeters)	165 (161, 170)	165 (162, 170)	0.27	-
BMI before pregnancy (median (IQR))	23.7 (21.7, 28.5)	22.7 (20.8, 26)	0.002 **	↑ in GDM
BMI > 25 before pregnancy (median (IQR))	46 (39.3%)	214 (31.5%)	0.12	-
BMI > 30 before pregnancy (median (IQR))	25 (21.4%)	75 (11%)	0.003 **	↑ in GDM
Smoking	21 (18%)	60 (8.8%)	0.004 **	↑ in GDM
Physical activity before pregnancy	0.143 (0, 0.714)	0.429 (0, 0.714)	0.43	-
Physical activity during pregnancy	0 (0, 0.429)	0 (0, 0.286)	0.08	-
Thyroid disease	13 (11.1%)	93 (13.7%)	0.54	-
Parity				
0	60 (51.3%)	347 (51%)	0.96	-
1	44 (37.6%)	253 (37.2%)	0.98	-
2	12 (10.3%)	69 (10.2%)	0.9	-
3	1 (0.9%)	9 (1.3%)	0.98	-
4	0 (0%)	2 (0.3%)	-	-
Planned pregnancy (*n*%)	103 (88%)	582 (85.7%)	0.6	-
Conception with ART (*n*%)	11 (9%)	48 (7.1%)	0.48	-

IQR: interquartile range, represented by the 25th percentile, median, and 75th percentile; *n*: number of participants; SD: standard deviation; MA: maternal age in years; BMI: body mass index calculated as weight in kilograms divided by height in meters squared (kg/m^2^). Thyroid disease includes conditions such as hypothyroidism, hyperthyroidism, and Hashimoto’s disease. Conception ART refers to conception through assisted reproductive technologies; the “↑” symbol indicates that values in the GDM group are higher than those in the non-GDM group; “**” denotes *p* < 0.01, and “***” denotes *p* < 0.001. Descriptive statistics include mean (SD), percentiles, and proportions.

**Table 2:** Pre-pregnancy amino acid intake and GDM risk.

Amino Acids	*p*-Value (aOR)	aOR (95% CI)
Histidine	0.67	0.88 (0.5, 1.54)
Isoleucine	0.69	0.93 (0.68, 1.28)
Leucine	0.54	0.94 (0.77, 1.13)
Lysine	0.77	0.97 (0.79, 1.18)
Methionine	0.73	0.88 (0.44, 1.75)
Phenylalanine	0.59	0.9 (0.63, 1.29)
Threonine	0.59	0.9 (0.61, 1.31)
Valine	0.69	0.94 (0.72, 1.23)
Alanine	0.49	0.89 (0.64, 1.22)
Cysteine	0.68	0.78 (0.24, 2.45)
Aspartic Acid	0.8	0.97 (0.8, 1.18)
Glutamic Acid	0.22	0.94 (0.86, 1.03)
Serine	0.54	0.91 (0.67, 1.21)
Tyrosine	0.44	0.85 (0.56, 1.27)
Tryptophane	0.74	0.79 (0.2, 3)
Proline	0.23	0.87 (0.69, 1.08)
Glycine	0.33	0.84 (0.58, 1.15)
Arginine	0.86	0.97 (0.74, 1.28)
EEAs%	0.19	1.1 (0.95, 1.28)
EAAs	0.87	0.99 (0.96, 1.03)
NEAAs	0.6	0.99 (0.95, 1.02)
BCAAs	0.63	0.97 (0.9, 1.06)
AAAs	0.54	0.94 (0.79, 1.12)

EEAs: histidine, isoleucine, leucine, lysine, methionine, phenylalanine, threonine, tryptophan, and valine; NEAAs: alanine, aspartic acid, glutamic acid, and serine; BCAAs: leucine, isoleucine, and valine; AAAs: phenylalanine, tyrosine, and tryptophan.

**Table 3:** Amino acid intake during pregnancy and GDM risk.

Amino Acids	*p*-Value (aOR)	aOR (95% CI)
Histidine	0.1	1.77 (0.89, 3.54)
Isoleucine	0.034 *	1.48 (1.03, 2.14)
Leucine	0.079	1.22 (0.97, 1.53)
Lysine	0.094	1.21 (0.96, 1.53)
Methionine	0.15	1.79 (0.8, 3.98)
Phenylalanine	0.03 *	1.6 (1.04,2.45)
Threonine	0.048 *	1.56 (1, 2.43)
Valine	0.075	1.31 (0.97, 1.77)
Alanine	0.1	1.38 (0.93, 2.05)
Cysteine	0.014 *	5.75 (1.42, 23.46)
Aspartic Acid	0.016 *	1.32 (1.05, 1.66)
Glutamic Acid	0.089	1.1 (0.98, 1.23)
Serine	0.15	1.25 (0.91, 1.71)
Tyrosine	0.16	1.38 (0.87, 2.2)
Tryptophan	0.15	2.91 (0.67, 12.71)
Proline	0.14	1.2 (0.93, 1.56)
Glycine	0.064	1.55 (0.96, 2.46)
Arginine	0.008 **	1.53 (1.11, 2.1)
EAAs%	0.21	1.1 (0.95, 1.28)
EAAs	0.02 *	1.04 (1, 1.09)
NEAAs	0.026 *	1.05 (1, 1.1)
BCAAs	0.061	1.09 (0.99, 1.2)
AAAs	0.08	1.2 (0.97, 1.47)

EAAs: histidine, isoleucine, leucine, lysine, methionine, phenylalanine, threonine, tryptophan, and valine; NEAAs: alanine, aspartic acid, glutamic acid, and serine; BCAAs: leucine, isoleucine, and valine; AAAs: phenylalanine, tyrosine, and tryptophane. “*” denotes *p* < 0.05, and “**” denotes *p* < 0.01.

## Data Availability

Not available due to ethical regulations.

## Supplementary Materials

The following supporting information can be downloaded at https://www.mdpi.com/article/10.3390/nu17010173/s1, Table S1: Difference in maternal macronutrient intake from Period B (during pregnancy) in comparison to Period A (pre-pregnancy); Table S2: Post hoc power analysis; Table S3: Quartiles of amino acid intakes; Table S4: Quartile cut points; Table S5: Confounders supplementary.
