# Supplementary material for: Relationship Between Amino Acid Intake in Maternal Diet and Risk of Gestational Diabetes Mellitus: Results from the BORN 2020 Pregnant Cohort in Northern Greece
Source: Nutrients. 2025 Jan 2;17(1):173. doi: 10.3390/nu17010173 (PMC11723356; doi:10.3390/nu17010173)
Supplement: Supplementary file 1 [file nutrients-17-00173-s001.zip › nutrients-3400127-supplementary.pdf]

Table S1. Difference in maternal macronutrient intake from period B (during pregnancy) in comparison to period A (pre-pregnancy).

Difference in maternal macronutrient intake	p-value (aOR)	aOR (95% CI)
Energy (B - A)	0.39	1 (0.99,1)
Protein (B - A)	0.007**	1 (1,1.01)
Protein % (B - A)	0.27	1.02 (0.98,1.07)
Vegetable protein (B - A)	0.044*	1.01 (0.99,1.03)
Animal protein - (B - A)	0.019*	1 (1,1.01)

B-A: indicates difference in intakes between the pregnancy (period B) and the pre-pregnancy (Period A) periods; A: period A (pre-pregnancy); B: period B (during pregnancy until mid-gestation before the OGTT)

Table S2. Post hoc power analysis.

Variable	p-value (aOR)	aOR (95% CI)	power (aOR)
Histidine – Period A	0.67	0.88 (0.5,1.54)	0.659
Isoleucine - Period A	0.69	0.93 (0.68,1.28)	0.237
Leucine - Period A	0.54	0.94 (0.77,1.13)	0.214
Lysine - Period A	0.77	0.97 (0.79,1.18)	0.09
Methionine - Period A	0.73	0.88 (0.44,1.75)	0.643
Phenylalanine - Period A	0.59	0.9 (0.63,1.29)	0.489
Threonine - Period A	0.59	0.9 (0.61,1.31)	0.522
Valine – Period A	0.69	0.94 (0.72,1.23)	0.187
Alanine – Period A	0.49	0.89 (0.64,1.22)	0.592
Cysteine – Period A	0.68	0.78 (0.24,2.45)	0.997
Aspartic acid – Period A	0.8	0.97 (0.8,1.18)	0.075
Glutamic acid – Period A	0.22	0.94 (0.86,1.03)	0.179
Serine – Period A	0.54	0.91 (0.67,1.21)	0.448
Tyrosine – Period A	0.44	0.85 (0.56,1.27)	0.883
Tryptophane – Period A	0.74	0.79 (0.2,3)	0.995
Proline – Period A	0.23	0.87 (0.69,1.08)	0.769
Glycine – Period A	0.33	0.84 (0.58,1.15)	0.912
Arginine – Period A	0.86	0.97 (0.74,1.28)	0.075
EAAs% - Period A	0.19	1.1 (0.95,1.28)	0.507
EAAs - Period A	0.87	0.99 (0.96,1.03)	0.05
NEAAs – Period A	0.6	0.99 (0.95,1.02)	0.054
BCAASs – Period A	0.63	0.97 (0.9,1.06)	0.069
AAAs – Period A	0.54	0.94 (0.79,1.12)	0.182
Histidine - Period B	0.1	1.77 (0.89,3.54)	1
Isoleucine – Period B	0.034*	1.48 (1.03,2.14)	1

Leucine – Period B	0.079	1.22 (0.97,1.53)	0.979
Lysine – Period B	0.094	1.21 (0.96,1.53)	0.975
Methionine – Period B	0.15	1.79 (0.8,3.98)	1
Phenylalanine – Period B	0.03*	1.6 (1.04,2.45)	1
Threonine – Period B	0.048*	1.56 (1,2.43)	1
Valine – Period B	0.075	1.31 (0.97,1.77)	1
Alanine – Period B	0.1	1.38 (0.93,2.05)	1
Cysteine – Period B	0.014*	5.75 (1.42,23.46)	1
Aspartic acid – Period B	0.016*	1.32 (1.05,1.66)	1
Glutamic acid – Period B	0.089	1.1 (0.98,1.23)	0.482
Serine – Period B	0.15	1.25 (0.91,1.71)	0.995
Tyrosine – Period B	0.16	1.38 (0.87,2.2)	1
Tryptophane – Period B	0.15	2.91 (0.67,12.71)	1
Proline – Period B	0.14	1.2 (0.93,1.56)	0.966
Glycine – Period B	0.064	1.55 (0.96,2.46)	1
Arginine – Period B	0.008**	1.53 (1.11,2.1)	1
EAAs % - Period B	0.21	1.1 (0.95,1.28)	0.492
EAAs – Period B	0.02*	1.04 (1,1.09)	0.161
NEAAs – Period B	0.026*	1.05 (1,1.1)	0.195
BCAASs – Period B	0.061	1.09 (0.99,1.2)	0.444
AAAs – Period B	0.08	1.2 (0.97,1.47)	0.957

B-A: indicates difference in intakes between the pregnancy (period B) and the pre-pregnancy (Period A) periods; A: period A (pre-pregnancy); B: period B (during pregnancy until mid gestation before the OGTT)

Table S3. Quartiles of amino acid intakes

**Variables**	**p-value (aOR)**	**aOR (95% CI)**	**power (aOR)**
Isoleucine – Period A_Q4`medium	0.54	0.81 (0.42,1.54)	0.979
Isoleucine – Period A_Q4`high	0.54	1.2 (0.66,2.22)	0.965
Isoleucine – Period A_Q4`very_high	0.36	1.37 (0.69,2.72)	1
Leucine – Period A_Q4`medium	0.77	0.9 (0.47,1.73)	0.491
Leucine – Period A_Q4`high	0.13	1.59 (0.88,2.94)	1
Leucine – Period A_Q4`very_high	0.53	1.25 (0.61,2.56)	0.995
Valine – Period A_Q4`medium	0.79	1.09 (0.58,2.04)	0.405

Valine – Period A_Q4`high	0.26	1.42 (0.77,2.65)	1
Valine – Period A_Q4`very_high	0.37	1.38 (0.67,2.83)	1
Lysine – Period A_Q4`medium	0.99	0.99 (0.53,1.86)	0.051
Lysine – Period A_Q4`high	0.53	1.21 (0.65,2.27)	0.977
Lysine – Period A_Q4`very_high	0.19	1.56 (0.79,3.07)	1
Methionine – Period A_Q4`medium	0.55	0.82 (0.43,1.55)	0.969
Methionine – Period A_Q4`high	0.45	1.26 (0.69,2.32)	0.996
Methionine – Period A_Q4`very_high	0.38	1.35 (0.69,2.66)	1
Phenylalanine – Period A_Q4`medium	0.7	0.88 (0.45,1.68)	0.705
Phenylalanine – Period A_Q4`high	0.12	1.61 (0.88,2.97)	1
Phenylalanine – Period A_Q4`very_high	0.41	1.34 (0.66,2.75)	1
Threonine – Period A_Q4`medium	0.9	0.96 (0.51,1.79)	0.128
Threonine – Period A_Q4`high	0.58	1.18 (0.64,2.2)	0.931
Threonine – Period A_Q4`very_high	0.32	1.41 (0.7,2.85)	1
Tryptophane – Period A_Q4`medium	0.94	0.97 (0.52,1.8)	0.077
Tryptophane – Period A_Q4`high	0.86	1.05 (0.56,1.99)	0.213
Tryptophane – Period A_Q4`very_high	0.19	1.57 (0.79,3.14)	1
Histidine – Period A_Q4`medium	1	0.99 (0.52,1.88)	0.05
Histidine – Period A_Q4`high	0.35	1.34 (0.72,2.53)	1
Histidine – Period A_Q4`very_high	0.096	1.78 (0.9,3.54)	1
Alanine – Period A_Q4`medium	0.31	1.37 (0.74,2.56)	1
Alanine – Period A_Q4`high	0.33	1.36 (0.73,2.57)	1
Alanine – Period A_Q4`very_high	0.33	1.42 (0.69,2.9)	1

Aspartic acid – Period A_Q4`medium	0.11	1.65 (0.89,3.1)	1
Aspartic acid – Period A_Q4`high	0.32	1.39 (0.72,2.69)	1
Aspartic acid – Period A_Q4`very_high	0.076	1.9 (0.93,3.89)	1
Glutamic acid – Period A_Q4`medium	0.64	1.16 (0.61,2.19)	0.853
Glutamic acid – Period A_Q4`high	0.22	1.48 (0.79,2.78)	1
Glutamic acid – Period A_Q4`very_high	0.37	1.39 (0.67,2.93)	1
Cysteine – Period A_Q4`medium	0.53	1.21 (0.65,2.26)	0.976
Cysteine – Period A_Q4`high	0.24	1.43 (0.78,2.67)	1
Cysteine – Period A_Q4`very_high	0.24	1.49 (0.76,2.9)	1
Glycine – Period A_Q4`medium	0.044*	1.87 (1.02,3.49)	1
Glycine – Period A_Q4`high	0.28	1.43 (0.74,2.81)	1
Glycine – Period A_Q4`very_high	0.14	1.7 (0.84,3.45)	1
Proline – Period A_Q4`medium	0.47	1.24 (0.67,2.31)	0.993
Proline – Period A_Q4`high	0.38	1.32 (0.71,2.47)	1
Proline – Period A_Q4`very_high	0.49	1.29 (0.62,2.66)	0.999
Serine – Period A_Q4`medium	0.84	1.06 (0.57,1.97)	0.247
Serine – Period A_Q4`high	0.92	1.03 (0.55,1.94)	0.094
Serine – Period A_Q4`very_high	0.27	1.47 (0.73,2.96)	1
Arginine – Period A_Q4`medium	0.34	1.34 (0.72,2.51)	1
Arginine – Period A_Q4`high	0.25	1.45 (0.77,2.76)	1
Arginine – Period A_Q4`very_high	0.15	1.64 (0.83,3.27)	1
Tyrosine – Period A_Q4`medium	0.96	0.98 (0.52,1.82)	0.064
Tyrosine – Period A_Q4`high	0.45	1.26 (0.69,2.32)	0.996

Tyrosine – Period A_Q4`very_high	0.55	1.23 (0.61,2.49)	0.988
EAAs – Period A_Q4`medium	0.13	1.6 (0.86,3.02)	1
EAAs – Period A_Q4`high	0.54	1.22 (0.63,2.38)	0.984
EAAs – Period A_Q4`very_high	0.048*	2 (1.01,4.05)	1
NEAAs – Period A_Q4`medium	0.43	1.28 (0.69,2.38)	0.998
NEAAs – Period A _Q4`high	0.51	1.24 (0.65,2.39)	0.992
NEAAs – Period A _Q4`very_high	0.15	1.66 (0.83,3.35)	1
EAAs% - Period A_Q4`medium	0.2	0.67 (0.36,1.21)	1
EAAs% - Period A_Q4`high	0.56	0.84 (0.46,1.49)	0.931
EAAs% - Period A_Q4`very_high	0.93	1.02 (0.59,1.77)	0.075
BCAASs – Period A_Q4`medium	0.88	0.95 (0.49,1.8)	0.171
BCAASs – Period A_Q4`high	0.25	1.42 (0.78,2.64)	1
BCAAS – Period A_Q4`very_high	0.36	1.38 (0.68,2.81)	1
AAAs – Period A_Q4`medium	0.81	0.92 (0.49,1.73)	0.325
AAAs – Period A_Q4`high	0.41	1.28 (0.7,2.37)	0.999
AAAs – Period A_Q4`very_high	0.49	1.28 (0.63,2.58)	0.999
Isoleucine – Period B_Q4`medium	0.001**	2.97 (1.54,5.97)	1
Isoleucine – Period B_Q4`high	0.042*	2.15 (1.03,4.62)	1
Isoleucine – Period B_Q4`very_high	p<0.001***	4.2 (1.91,9.58)	1
Leucine – Period B_Q4`medium	0.015*	2.25 (1.18,4.42)	1
Leucine – Period B_Q4`high	0.01*	2.47 (1.25,5.02)	1
Leucine – Period B_Q4`very_high	0.009**	2.89 (1.32,6.49)	1
Valine – Period B_Q4`medium	0.015*	2.18 (1.17,4.18)	1

Valine – Period B_Q4`high	0.14	1.67 (0.84,3.38)	1
Valine – Period B_Q4`very_high	0.027*	2.4 (1.11,5.29)	1
Lysine – Period B_Q4`medium	0.021*	2.1 (1.12,4.02)	1
Lysine – Period B_Q4`high	0.12	1.71 (0.87,3.41)	1
Lysine – Period B_Q4`very_high	0.021*	2.37 (1.14,5.03)	1
Methionine – Period B_Q4`medium	0.016*	2.2 (1.17,4.28)	1
Methionine – Period B_Q4`high	0.056	1.95 (0.99,3.94)	1
Methionine – Period B_Q4`very_high	0.005**	2.95 (1.38,6.44)	1
Phenylalanine – Period B_Q4`medium	0.009**	2.37 (1.25,4.61)	1
Phenylalanine – Period B_Q4`high	0.046*	2.05 (1.02,4.2)	1
Phenylalanine – Period B_Q4`very_high	0.004**	3.2 (1.46,7.16)	1
Threonine – Period B_Q4`medium	0.007**	2.39 (1.28,4.62)	1
Threonine – Period B_Q4`high	0.12	1.75 (0.87,3.6)	1
Threonine – Period B_Q4`very_high	0.009**	2.8 (1.31,6.16)	1
Tryptophane – Period B_Q4`medium	0.04*	1.96 (1.04,3.79)	1
Tryptophane – Period B_Q4`high	0.061	1.9 (0.97,3.8)	1
Tryptophane – Period B_Q4`very_high	0.029*	2.35 (1.09,5.16)	1
Histidine – Period B_Q4`medium	0.69	1.13 (0.61,2.11)	0.709
Histidine – Period B_Q4`high	0.32	1.38 (0.72,2.67)	1
Histidine – Period B_Q4`very_high	0.12	1.76 (0.85,3.68)	1
Alanine – Period B_Q4`medium	0.37	1.31 (0.71,2.43)	1
Alanine – Period B_Q4`high	0.5	1.25 (0.64,2.43)	0.995
Alanine – Period B_Q4`very_high	0.23	1.57 (0.75,3.31)	1

Aspartic acid – Period B_Q4`medium	0.031*	2.01 (1.07,3.86)	1
Aspartic acid – Period B_Q4`high	0.19	1.6 (0.79,3.27)	1
Aspartic acid – Period B_Q4`very_high	0.009**	2.8 (1.31,6.12)	1
Glutamic acid – Period B_Q4`medium	0.31	1.37 (0.74,2.57)	1
Glutamic acid – Period B_Q4`high	0.21	1.52 (0.78,2.98)	1
Glutamic acid – Period B_Q4`very_high	0.4	1.4 (0.63,3.15)	1
Cysteine – Period B_Q4`medium	0.092	1.72 (0.92,3.27)	1
Cysteine – Period B_Q4`high	0.044*	1.93 (1.02,3.72)	1
Cysteine – Period B_Q4`very_high	0.042*	2.06 (1.03,4.2)	1
Glycine – Period B_Q4`medium	0.93	0.97 (0.53,1.77)	0.088
Glycine – Period B_Q4`high	0.79	0.91 (0.47,1.76)	0.435
Glycine – Period B_Q4`very_high	0.28	1.46 (0.73,2.92)	1
Proline – Period B_Q4`medium	0.04*	1.96 (1.04,3.77)	1
Proline – Period B_Q4`high	0.031*	2.08 (1.07,4.14)	1
Proline – Period B_Q4`very_high	0.074	2.08 (0.93,4.69)	1
Serine – Period B_Q4`medium	0.19	1.51 (0.82,2.82)	1
Serine – Period B_Q4`high	0.32	1.39 (0.72,2.69)	1
Serine – Period B_Q4`very_high	0.23	1.59 (0.75,3.42)	1
Arginine – Period B_Q4`medium	0.41	1.29 (0.69,2.44)	0.999
Arginine – Period B_Q4`high	0.35	1.37 (0.7,2.66)	1
Arginine – Period B_Q4`very_high	0.024*	2.26 (1.11,4.64)	1
Tyrosine – Period B_Q4`medium	0.38	1.33 (0.7,2.59)	1
Tyrosine – Period B_Q4`high	0.023*	2.13 (1.12,4.16)	1

Tyrosine – Period B_Q4`very_high	0.058	2.11 (0.98,4.62)	1
EAAs – Period B_Q4`medium	0.007**	2.41 (1.28,4.67)	1
EAAs – Period B_Q4`high	0.11	1.78 (0.88,3.65)	1
EAAs – Period B_Q4`very_high	0.012*	2.68 (1.25,5.85)	1
NEAAs – Period B_Q4`medium	0.039*	1.93 (1.04,3.65)	1
NEAAs – Period B_Q4`high	0.3	1.44 (0.72,2.89)	1
NEAAs – Period B_Q4`very_high	0.073	2.01 (0.93,4.36)	1
EAAs% - Period B_Q4`medium	0.77	0.91 (0.49,1.68)	0.451
EAAs% - Period B_Q4`high	0.71	1.11 (0.61,2.02)	0.6
EAAs% - Period B_Q4`very_high	0.3	1.34 (0.76,2.4)	1
BCAASs – Period B_Q4`medium	0.002**	2.87 (1.49,5.76)	1
BCAASs – Period B_Q4`high	0.007**	2.7 (1.34,5.65)	1
BCAASs – Period B_Q4`very_high	0.005**	3.22 (1.43,7.46)	1
AAAs – Period B_Q4`medium	0.023*	2.12 (1.12,4.12)	1
AAAs – Period B _Q4`high	0.04*	2.06 (1.04,4.16)	1
AAAs – Period B _Q4`very_high	0.017*	2.59 (1.19,5.76)	1

B-A: indicates difference in intakes between the pregnancy (period B) and the pre-pregnancy (Period A) periods; A: period A (pre-pregnancy); B: period B (during pregnancy until mid gestation before the OGTT)

Table S4. Quartile cut points

AminoAcid	Cut1	Cut2	Cut3	Cut4
energy - (B - A)	[-4294.75,-216.16)	[-216.16, -8.66)	[-8.66, 170.19)	[170.19,2134.18]
protein - (B - A)	[-274.726,-6.288)	[-6.288,-0.125)	[-0.125, 5.864)	[5.864,92.143]
protein % - (B - A)	[-1.37e+01,-1.247791)	[-1.25e+00, 0.000415)	[4.15e-04, 1.390502)	[1.39e+00,11.932303]

veget. protein - (B - A)	[-81.341,-2.337)	[-2.337,-0.115)	[-0.115, 2.271)	[2.271,59.343]
aniMaternal agel protein - (B - A)	[-1.93e+02,-6.06e+00)	[-6.06e+00, 3.55e-15)	[3.55e-15, 5.05e+00)	[5.05e+00, 9.21e+01]
isoleucine - Period A	[0.143, 1.95)	[1.953, 2.38)	[2.383, 2.89)	[2.889,15.64]
leucine - Period A	[0.214, 3.18)	[3.183, 3.89)	[3.887, 4.74)	[4.740,25.08]
valine - Period A	[0.143, 2.24)	[2.241, 2.76)	[2.763, 3.38)	[3.378,17.46]
lysine - Period A	[0.143, 2.67)	[2.669, 3.32)	[3.322, 4.07)	[4.070,22.42]
methionine - Period A	[0.0714,0.782)	[0.7824,0.974)	[0.9738,1.194)	[1.1937,6.858]
phenylalanine - Period A	[0.214, 1.78)	[1.780, 2.16)	[2.158, 2.61)	[2.608,13.97]
threonine - Period A	[0.143, 1.64)	[1.637, 2.00)	[1.998, 2.43)	[2.428,13.32]
tryptophane - Period A	[0.0714,0.409)	[0.4090,0.518)	[0.5181,0.643)	[0.6433,3.375]
histidine - Period A	[0.0714,0.964)	[0.9643,1.202)	[1.2021,1.453)	[1.4525,9.072]
alanine - Period A	[0.143, 1.97)	[1.972, 2.36)	[2.362, 2.84)	[2.837,16.55]
aspar. acid - Period A	[0.214, 3.45)	[3.452, 4.20)	[4.204, 4.97)	[4.975,28.22]
glutamic acid - Period A	[0.857, 7.74)	[7.740, 9.38)	[9.381,11.30)	[11.298,66.70]
cysteine - Period A	[0.0557,0.371)	[0.3714,0.451)	[0.4514,0.560)	[0.5595,4.010]
glycine - Period A	[0.143, 1.44)	[1.445, 1.74)	[1.735, 2.09)	[2.092,13.87]
proline - Period A	[0.357, 2.66)	[2.656, 3.32)	[3.316, 4.08)	[4.079,19.80]
serine - Period A	[0.143, 2.05)	[2.046, 2.53)	[2.526, 3.12)	[3.116,14.66]
arginine - Period A	[0.143, 2.06)	[2.062, 2.50)	[2.497, 3.05)	[3.052,19.39]
tyrosine - Period A	[0.0714, 1.46)	[1.4560, 1.80)	[1.7990, 2.20)	[2.1973,11.20]
EAAs - Period A	[0.0, 17.8)	[17.8, 21.5)	[21.5, 25.8)	[25.8,156.8]
NEAAs - Period A	[0.0, 16.6)	[16.6, 20.0)	[20.0, 24.0)	[24.0,153.6]
EAAs% - Period A	[34.4,41.2)	[41.2,42.1)	[42.1,42.8)	[42.8,45.6]
BCAASs - Period A	[0.50, 7.36)	[7.36, 9.05)	[9.05,10.98)	[10.98,58.18]
AAAs – Period A	[0.357, 3.64)	[3.643, 4.49)	[4.492, 5.45)	[5.455,28.55]
isoleucine - Period B	[0.129,1.93)	[1.933,2.39)	[2.393,2.86)	[2.862,6.72]
leucine - Period B	[0.157, 3.18)	[3.177, 3.89)	[3.887, 4.66)	[4.659,11.54]
valine - Period B	[0.129,2.23)	[2.231,2.76)	[2.756,3.33)	[3.330,8.65]
lysine - Period B	[0.0143, 2.65)	[2.6479, 3.33)	[3.3310, 4.00)	[4.0048,10.51]
methionine - Period B	[0.0143,0.762)	[0.7621,0.966)	[0.9657,1.174)	[1.1741,2.958]
phenylalanine - Period B	[0.129,1.77)	[1.770,2.16)	[2.160,2.58)	[2.582,5.97]
threonine - Period B	[0.114,1.63)	[1.627,1.99)	[1.987,2.40)	[2.402,5.81]

tryptophane - Period B	[0.0143,0.404)	[0.4043,0.507)	[0.5067,0.628)	[0.6276,1.712]
histidine - Period B	[0.0143,0.95)	[0.9502,1.19)	[1.1854,1.44)	[1.4383,3.67]
alanine - Period B	[0.129,1.94)	[1.944,2.35)	[2.350,2.80)	[2.801,6.59]
aspar. acid - Period B	[0.129, 3.42)	[3.422, 4.15)	[4.148, 4.95)	[4.947,12.23]
glutamic acid - Period B	[0.943, 7.63)	[7.626, 9.32)	[9.316,11.06)	[11.057,23.95]
cysteine - Period B	[0.0143,0.349)	[0.3486,0.444)	[0.4438,0.544)	[0.5438,1.691]
glycine - Period B	[0.129,1.41)	[1.407,1.71)	[1.706,2.06)	[2.062,5.28]
proline - Period B	[0.286, 2.64)	[2.640, 3.34)	[3.342, 4.01)	[4.006,10.99]
serine - Period B	[0.143,2.04)	[2.040,2.55)	[2.550,3.11)	[3.108,8.94]
arginine - Period B	[0.129,2.01)	[2.012,2.48)	[2.480,2.99)	[2.995,9.79]
Tyrosine - Period B	[0.114,1.44)	[1.442,1.79)	[1.787,2.17)	[2.170,5.68]
EAAs – Period B	[1.07,17.4)	[17.39,21.5)	[21.53,25.7)	[25.71,64.2]
NEAAs – Period B	[1.76,16.3)	[16.29,19.9)	[19.91,24.0)	[24.01,55.6]
EAAs % - Period B	[27.3,41.2)	[41.2,42.2)	[42.2,42.9)	[42.9,46.2]
BCAASs – Period B	[0.414, 7.37)	[7.371, 9.01)	[9.013,10.83)	[10.832,26.73]
AAAs – Period B	[0.805, 3.68)	[3.681, 4.48)	[4.482, 5.36)	[5.360,11.98]

B-A: indicates difference in intakes between the pregnancy (period B) and the pre-pregnancy (Period A) periods; A: period A (pre-pregnancy); B: period B (during pregnancy until mid-gestation before the OGTT)

Table S5: Confounders supplementary

Exposure	Variable Name	OR	CI_low	CI_high	P value	Estimate	Std. Error	z value
isoleucine - A	isoleucine - A	0.93972272	0.6862967	1.28247001	0.69277039	-0.06217043	0.15735435	-0.39509824
isoleucine - A	energy - A	1.00024637	0.99962184	1.00085124	0.43104428	0.00024634	0.00031286	0.78740592
isoleucine - A	BMI pre-pregnancy	1.06953556	1.02917948	1.11104677	0.00055369	0.06722449	0.01946651	3.45334149
isoleucine - A	ART	0.78037773	0.34364177	1.64803535	0.53262481	-0.2479772	0.39739667	-0.62400422
isoleucine - A	Physical activity A	0.89716438	0.51359923	1.54803142	0.6992213	-0.10851618	0.28085943	-0.38637185
isoleucine - A	Maternal age	1.10346407	1.05422748	1.15672282	3.1049E-05	0.09845439	0.02363493	4.16563046
isoleucine - A	Thyroid disease	0.68394579	0.34596023	1.25787769	0.24534861	-0.37987662	0.32699457	-1.1617215
isoleucine - A	Smoking	2.17625224	1.1957035	3.85182393	0.00886958	0.77760424	0.29713114	2.61704052
isoleucine - A	Parity	0.83267906	0.54229735	1.27611045	0.40069197	-0.18310699	0.21788438	-0.84038605
isoleucine - A	supplements	3148828.72	6.0547E-05	3.2001E+40	0.97425991	14.9625411	463.724374	0.03226602
isoleucine - A	Weight gain	0.96335436	0.89141321	1.02822795	0.31303866	-0.03733396	0.03700585	-1.0088665
leucine - A	leucine - A	0.94338419	0.77780512	1.13868002	0.54466618	-0.05828166	0.09621056	-0.60577197
leucine - A	energy - A	1.00030039	0.99967338	1.00090863	0.33940968	0.00030034	0.00031438	0.95533231
leucine - A	BMI pre-pregnancy	1.06955863	1.02919402	1.11108997	0.00055397	0.06724606	0.01947353	3.45320338
leucine - A	ART	0.77590611	0.34134398	1.64002875	0.52367081	-0.25372376	0.39787498	-0.6376972
leucine - A	Physical activity A	0.89622545	0.51304294	1.54645928	0.69648108	-0.10956328	0.28087745	-0.39007501
leucine - A	Maternal age	1.10357054	1.05435004	1.15681026	3.0251E-05	0.09855087	0.02362442	4.1715669
leucine - A	Thyroid disease	0.6791144	0.34323648	1.25000182	0.23725415	-0.38696569	0.32741596	-1.18187791
leucine - A	Smoking	2.17688058	1.19649931	3.85200844	0.00880936	0.77789293	0.29697768	2.61936496
leucine - A	Parity	0.83479114	0.54363031	1.27951703	0.40735641	-0.18057372	0.21793819	-0.82855473
leucine - A	supplements	3168302.87	6.1549E-05	2.964E+40	0.97423788	14.9687066	463.518686	0.03229364
leucine - A	Weight gain	0.96296964	0.89098206	1.0279186	0.30863434	-0.0377334	0.03706286	-1.01809179
valine - A	valine - A	0.94802981	0.72370803	1.23677887	0.69399501	-0.05336933	0.13564818	-0.39343935
valine - A	energy - A	1.00024798	0.99961548	1.00085973	0.43363219	0.00024794	0.00031666	0.78299135
valine - A	BMI pre-pregnancy	1.06943129	1.02908828	1.11092115	0.00056189	0.067127	0.01946065	3.44937119
valine - A	ART	0.7786071	0.34258768	1.64546	0.52928532	-0.25024873	0.39779015	-0.62909735
valine - A	Physical activity A	0.8957662	0.51269305	1.54595028	0.69522509	-0.11007584	0.28096758	-0.39177415
valine - A	Maternal age	1.10337871	1.05416892	1.15660731	3.1223E-05	0.09837702	0.02362358	4.16435743
valine - A	Thyroid disease	0.6835785	0.34569241	1.25750835	0.24486014	-0.38041378	0.32711821	-1.1629245
valine - A	Smoking	2.17751625	1.19614425	3.85485497	0.00884343	0.77818489	0.29723856	2.61804827
valine - A	Parity	0.83306245	0.54246221	1.27692885	0.40206252	-0.18264667	0.21797018	-0.83794334

valine - A	supplements	3147189.62	6.0262E-05	3.282E+40	0.97426537	14.9620204	463.806696	0.03225917
valine - A	Weight gain	0.96339643	0.89142568	1.02830367	0.31387252	-0.03729029	0.03702631	-1.00712955
lysine - A	lysine - A	0.97096822	0.79239136	1.18786065	0.77286808	-0.02946154	0.10207536	-0.28862537
lysine - A	energy - A	1.00021388	0.99962048	1.00078644	0.47083748	0.00021386	0.00029657	0.72111709
lysine - A	BMI pre-pregnancy	1.0695125	1.0291521	1.1110218	0.00055625	0.06720294	0.01946728	3.4520962
lysine - A	ART	0.78088348	0.34377799	1.64944239	0.53381813	-0.24732934	0.39751532	-0.62218819
lysine - A	Physical activity A	0.89681374	0.51331775	1.54768361	0.69827572	-0.10890709	0.28094244	-0.3876491
lysine - A	Maternal age	1.10314578	1.05398474	1.15631241	3.1962E-05	0.09816589	0.02360316	4.15901492
lysine - A	Thyroid disease	0.68636923	0.34743709	1.26154383	0.24926304	-0.37633956	0.3266436	-1.15214125
lysine - A	Smoking	2.1729239	1.19369351	3.84655141	0.00902294	0.77607368	0.29721144	2.61118372
lysine - A	Parity	0.83166384	0.54162143	1.27459243	0.39759277	-0.18432696	0.21789904	-0.84592825
lysine - A	supplements	3136384.58	5.977E-05	3.454E+40	0.97427681	14.9585813	463.906489	0.03224482
lysine - A	Weight gain	0.96348644	0.89151171	1.02838238	0.31499337	-0.03719687	0.03701919	-1.00479956
methionine - A	methionine -	0.88992942	0.44747708	1.75425708	0.7343004	-0.11661313	0.34357534	-0.33941065
methionine - A	energy - A`	1.00022686	0.99962836	1.00080726	0.44946087	0.00022683	0.00029991	0.75631414
methionine - A	BMI pre-pregnancy	1.06937141	1.02903096	1.11085494	0.00056752	0.06707101	0.01945959	3.44668205
methionine - A	ART	0.78031791	0.34353633	1.6482377	0.53261319	-0.24805387	0.39750826	-0.62402191
methionine - A	Physical activity A	0.89746566	0.51374052	1.54862176	0.70013412	-0.10818042	0.28088637	-0.38513943
methionine - A	Maternal age	1.1031301	1.05398154	1.15628431	3.1909E-05	0.09815169	0.02359759	4.15939475
methionine - A	Thyroid disease	0.68420998	0.34595813	1.25892874	0.24613922	-0.37949042	0.32720954	-1.15977796
methionine - A	Smoking	2.17787464	1.19608843	3.85615196	0.00885114	0.77834947	0.29733518	2.61775105
methionine - A	Parity	0.83219308	0.54195739	1.27543455	0.3992457	-0.1836908	0.21790928	-0.84296915
methionine - A	supplements	3133473.28	5.9776E-05	2.9543E+40	0.97427513	14.9576526	463.847292	0.03224693
methionine - A	Weight gain	0.96338576	0.89139377	1.02831162	0.31390159	-0.03730137	0.03703954	-1.00706907
phenylalanine - A	phenylalanine - A	0.90770836	0.63173169	1.29740193	0.59269584	-0.09683214	0.18101716	-0.53493351
phenylalanine - A	energy - A	1.00028714	0.99964532	1.00090846	0.37185494	0.00028709	0.00032149	0.89300417
phenylalanine - A	BMI pre-pregnancy	1.06942345	1.02907665	1.11092757	0.00056443	0.06711967	0.01946539	3.44815378
phenylalanine - A	ART	0.77754839	0.34214692	1.64313615	0.52701372	-0.2516094	0.39775667	-0.63257116
phenylalanine - A	Physical activity A	0.89634329	0.51312282	1.54663059	0.69681533	-0.1094318	0.28086585	-0.38962302
phenylalanine - A	Maternal age	1.10372881	1.05445163	1.15704253	3.0071E-05	0.09869427	0.02365106	4.17293164
phenylalanine - A	Thyroid disease	0.68105822	0.34431993	1.25314961	0.24049939	-0.38410748	0.32725105	-1.17373948
phenylalanine - A	Smoking	2.17509815	1.19557426	3.84865754	0.00887483	0.77707379	0.29695138	2.61683842
phenylalanine - A	Parity	0.833875	0.54308338	1.27796826	0.40439748	-0.18167177	0.21788586	-0.83379329
phenylalanine - A	supplements	3166023.19	6.1488E-05	3.081E+40	0.97423996	14.9679868	463.533898	0.03229103
phenylalanine - A	Weight gain	0.96319628	0.89129308	1.02803774	0.31069089	-0.03749807	0.03698861	-1.0137734

threonine - A	threonine - A	0.90399006	0.61938445	1.31403907	0.59281955	-0.10093691	0.1887537	-0.53475461
threonine - A	energy - A	1.00028265	0.99965359	1.00089164	0.36970828	0.00028261	0.00031506	0.89701994
threonine - A	BMI pre-pregnancy	1.0695672	1.02920306	1.11109247	0.0005524	0.06725408	0.01947154	3.45396781
threonine - A	ART	0.77787672	0.34241363	1.64337247	0.52753622	-0.25118723	0.39759191	-0.63177147
threonine - A	Physical activity A	0.89709871	0.51361034	1.54778751	0.698982	-0.10858937	0.280814	-0.38669502
threonine - A	Maternal age	1.10365722	1.05441612	1.15691475	2.9981E-05	0.09862941	0.02363165	4.17361418
threonine - A	Thyroid disease	0.68173394	0.34478545	1.25396528	0.24145404	-0.38311582	0.32706919	-1.17136015
threonine - A	Smoking	2.18382849	1.19983671	3.86543756	0.00857458	0.78107953	0.29715048	2.62856562
threonine - A	Parity	0.83469278	0.54351233	1.27945828	0.40714483	-0.18069155	0.21798205	-0.82892856
threonine - A	supplements	3168592.31	6.1534E-05	3.1916E+40	0.97423914	14.968798	463.544256	0.03229206
threonine - A	Weight gain	0.96316956	0.89117278	1.02811111	0.31120419	-0.03752581	0.03705526	-1.01269852
tryptophane - A	tryptophane - A	0.79550808	0.20662543	3.00545267	0.73608373	-0.22877428	0.6787668	-0.337044
tryptophane - A	energy - A	1.00023426	0.99959957	1.00084969	0.46157847	0.00023423	0.00031814	0.73625016
tryptophane - A	BMI pre-pregnancy	1.06938197	1.02905031	1.11085666	0.00056483	0.06708088	0.01945522	3.44796347
tryptophane - A	ART	0.78014649	0.34330172	1.64837307	0.53245541	-0.24827356	0.3977072	-0.62426219
tryptophane - A	Physical activity A	0.89815059	0.51410852	1.54982803	0.70216321	-0.10741753	0.28090212	-0.38240199
tryptophane - A	Maternal age	1.10298091	1.05386054	1.15610165	3.2439E-05	0.09801644	0.0235864	4.15563275
tryptophane - A	Thyroid disease	0.68509821	0.3466277	1.25973753	0.24727501	-0.37819308	0.32687571	-1.15699352
tryptophane - A	Smoking	2.17996141	1.19685191	3.8610634	0.00880457	0.77930718	0.29749649	2.61955081
tryptophane - A	Parity	0.83232919	0.54199777	1.27580274	0.39978254	-0.18352726	0.21796336	-0.84200967
tryptophane - A	supplements	3140812.75	6.0075E-05	3.1874E+40	0.97426953	14.9599922	463.818795	0.03225396
tryptophane - A	Weight gain	0.96335328	0.89128289	1.02835469	0.3141296	-0.03733508	0.03709048	-1.00659466
histidine - A	histidine - A	0.88801868	0.5089766	1.54201641	0.66731499	-0.1187625	0.27629729	-0.42983594
histidine - A	energy - A	1.00025198	0.99963795	1.00084656	0.4125665	0.00025194	0.00030748	0.81938552
histidine - A	BMI pre-pregnancy	1.06925494	1.02891561	1.11073656	0.0005801	0.06696209	0.01946148	3.44075108
histidine - A	ART	0.77872481	0.34268411	1.6454791	0.52946074	-0.25009755	0.39771923	-0.62882941
histidine - A	Physical activity A	0.8978368	0.51396718	1.54920346	0.70120806	-0.10776697	0.28086973	-0.38369022
histidine - A	Maternal age	1.10308071	1.05394728	1.15621278	3.1988E-05	0.09810691	0.02359003	4.15882978
histidine - A	Thyroid	0.68334898	0.34566188	1.25679095	0.24426208	-0.3807496	0.32699219	-1.16439968
histidine - A	Smoking	2.18334152	1.19905083	3.86602379	0.00863879	0.78085651	0.29735277	2.62602736
histidine - A	Parity	0.83267123	0.54231503	1.27607686	0.40063607	-0.18311639	0.21786971	-0.84048578
histidine - A	supplements	3149153.01	6.0622E-05	3.0543E+40	0.97425792	14.9626441	463.691784	0.03226851
histidine - A	Weight gain	0.96317504	0.89112109	1.02815729	0.31165841	-0.03752012	0.03708444	-1.01174831
alanine - A	alanine - A	0.89592998	0.64918003	1.22420059	0.48641444	-0.10989302	0.15788705	-0.69602298
alanine - A	energy - A	1.00032214	0.99969762	1.00092932	0.30399093	0.00032209	0.00031334	1.02791263

alanine - A	BMI pre-pregnancy	1.06976033	1.02932953	1.11136656	0.00054501	0.06743463	0.01950332	3.45759834
alanine - A	ART	0.77817345	0.34273165	1.64311493	0.52786621	-0.25080584	0.39730572	-0.63126662
alanine - A	Physical activity A	0.89504549	0.51239911	1.54437961	0.69299287	-0.11088074	0.28085522	-0.39479678
alanine - A	Maternal age	1.10365781	1.05445396	1.1568653	2.9513E-05	0.09862995	0.02361152	4.17719538
alanine - A	Thyroid disease	0.68021295	0.34413298	1.25082413	0.23849465	-0.38534937	0.32691142	-1.17875775
alanine - A	Smoking	2.1912597	1.20402341	3.87870625	0.00828797	0.78447658	0.2971381	2.64010773
alanine - A	Parity	0.83763571	0.54529613	1.2843169	0.41662257	-0.17717199	0.21811295	-0.81229468
alanine - A	supplements	3190770.22	6.2795E-05	3.0583E+40	0.97421312	14.9757729	463.292047	0.03232469
alanine - A	Weight gain	0.96288598	0.89092534	1.02776828	0.3071055	-0.03782027	0.03703098	-1.02131441
aspartic acid - A	aspartic acid - A	0.9768422	0.80959819	1.18122112	0.80298109	-0.02343015	0.09391186	-0.24949089
aspartic acid - A	energy - A	1.00021095	0.99958156	1.00082136	0.5036275	0.00021093	0.00031539	0.66879305
aspartic acid - A	BMI pre-pregnancy	1.06931857	1.02898824	1.11078499	0.00057088	0.06702159	0.01945425	3.44508651
aspartic acid - A	ART	0.78331928	0.3451492	1.65323818	0.53853356	-0.2442149	0.39707671	-0.61503205
aspartic acid - A	Physical activity A	0.89849677	0.51434587	1.55030047	0.70313918	-0.10703217	0.28086068	-0.38108635
aspartic acid - A	Maternal age	1.10332883	1.05404597	1.15664002	3.2393E-05	0.09833182	0.02366045	4.15595783
aspartic acid - A	Thyroid disease	0.68807237	0.3485573	1.26362198	0.25180233	-0.37386126	0.3262364	-1.14598265
aspartic acid - A	Smoking	2.17255256	1.1935852	3.84547978	0.00902583	0.77590277	0.29715845	2.61107425
aspartic acid - A	Parity	0.82998175	0.54078697	1.2713632	0.3918789	-0.18635157	0.21764578	-0.85621494
aspartic acid - A	supplements	3130931.46	5.9787E-05	3.1156E+40	0.97427631	14.9568411	463.843373	0.03224546
aspartic acid - A	Weight gain	0.96344212	0.89152416	1.02828885	0.31389947	-0.03724287	0.03698128	-1.00707347
glutamic acid - A	glutamic acid - A	0.94935936	0.86909748	1.03017357	0.21983917	-0.05196788	0.04235513	-1.2269559
glutamic acid - A	energy - A	1.00049849	0.99982593	1.00115089	0.13907089	0.00049837	0.0003369	1.47925981
glutamic acid - A	BMI pre-pregnancy	1.06945954	1.02906187	1.11104921	0.00057308	0.06715342	0.01949841	3.44404635
glutamic acid - A	ART	0.76838022	0.33770331	1.62593776	0.50842565	-0.26347059	0.39841848	-0.6612911
glutamic acid - A	Physical activity A	0.89516669	0.51235085	1.54492012	0.69346844	-0.11074533	0.28097075	-0.39415252
glutamic acid - A	Maternal age	1.10413304	1.05480785	1.15748629	2.8343E-05	0.09906045	0.02366253	4.18638529
glutamic acid - A	Thyroid disease	0.66724311	0.33675021	1.22977246	0.2175634	-0.40460081	0.32813458	-1.23303314
glutamic acid - A	Smoking	2.21757865	1.21950633	3.92367954	0.00729312	0.7964159	0.29682071	2.68315476
glutamic acid - A	Parity	0.84523639	0.55013221	1.29638639	0.44105723	-0.16813894	0.21824629	-0.77040914
glutamic acid - A	supplements	3287256.89	6.9362E-05	2.4921E+40	0.97408843	15.005564	461.979023	0.03248105
glutamic acid - A	Weight gain	0.9609857	0.88871069	1.02631901	0.28681119	-0.03979575	0.03736188	-1.06514307
cysteine - A	cysteine - A	0.7895671	0.24484402	2.45890178	0.67936955	-0.23627045	0.57163554	-0.41332359
cysteine - A	energy - A	1.00023055	0.99968218	1.00076807	0.40402749	0.00023053	0.00027626	0.83444994
cysteine - A	BMI pre-pregnancy	1.06904105	1.02870178	1.11052306	0.00060436	0.06676203	0.01946613	3.42965188
cysteine - A	ART	0.78307347	0.34500348	1.65287651	0.53806356	-0.24452875	0.39712736	-0.6157439

cysteine - A	Physical activity A	0.90454282	0.51726773	1.56229996	0.72143207	-0.10032563	0.28138241	-0.35654551
cysteine - A	Maternal age	1.1032061	1.05405822	1.15636137	3.1459E-05	0.09822058	0.02359574	4.16264059
cysteine - A	Thyroid disease	0.68450525	0.34635963	1.25837739	0.24608169	-0.37905897	0.32679773	-1.15991924
cysteine - A	Smoking	2.19081088	1.20184466	3.88286447	0.00846318	0.78427174	0.29786125	2.63301033
cysteine - A	Parity	0.83248864	0.54223119	1.27568585	0.39998724	-0.18333571	0.21783046	-0.84164402
cysteine - A	supplements	3150189.04	6.0766E-05	3.0176E+40	0.97425515	14.962973	463.652012	0.03227199
cysteine - A	Weight gain	0.96285355	0.89075828	1.02789567	0.30790388	-0.03785396	0.03712519	-1.01963019
glycine - A	glycine - A	0.84701712	0.58218043	1.15950095	0.32884423	-0.16603437	0.17003974	-0.97644451
glycine - A	energy - A	1.00035136	0.9998032	1.00089759	0.20684866	0.0003513	0.00027831	1.26227831
glycine - A	BMI pre-pregnancy	1.07074845	1.0301052	1.11264301	0.00048837	0.06835789	0.01960333	3.4870554
glycine - A	ART	0.79287126	0.34952936	1.67226084	0.55856948	-0.23209442	0.39676455	-0.58496764
glycine - A	Physical activity A	0.89581309	0.5128137	1.54571718	0.6952619	-0.1100235	0.2808697	-0.39172434
glycine - A	Maternal age	1.10329864	1.05415779	1.15643062	3.0762E-05	0.09830446	0.02358694	4.16775019
glycine - A	Thyroid disease	0.68429216	0.34677879	1.25638393	0.24464811	-0.37937032	0.32607438	-1.16344721
glycine - A	Smoking	2.19434998	1.20594693	3.88442964	0.00816746	0.78588586	0.29711372	2.64506753
glycine - A	Parity	0.83758641	0.54556022	1.28353646	0.41587276	-0.17723085	0.21783472	-0.81360241
glycine - A	supplements	3228426.74	6.5038E-05	2.7599E+40	0.97416787	14.9875055	462.842493	0.03238144
glycine - A	Weight gain	0.96225385	0.89047546	1.0268636	0.29695198	-0.03847699	0.03689097	-1.04299203
proline - A	proline - A	0.87363757	0.69564063	1.08674784	0.23422421	-0.13508967	0.11356389	-1.1895477
proline - A	energy - A	1.00046782	0.99982011	1.00109598	0.14972222	0.00046771	0.00032468	1.44051334
proline - A	BMI pre-pregnancy	1.07122022	1.03058442	1.11312126	0.0004466	0.06879839	0.0195957	3.51089274
proline - A	ART	0.76981257	0.33812906	1.62951065	0.51167754	-0.26160821	0.39865455	-0.65622783
proline - A	Physical activity A	0.88607038	0.50666536	1.53041973	0.66731866	-0.1209589	0.28141044	-0.42983089
proline - A	Maternal age	1.10337794	1.05415427	1.15660688	3.1303E-05	0.09837633	0.02362673	4.16377201
proline - A	Thyroid disease	0.66565732	0.33599707	1.22701321	0.21487584	-0.40698028	0.32813866	-1.24026919
proline - A	Smoking	2.17189129	1.19407781	3.84287579	0.00899173	0.77559835	0.29689472	2.61236829
proline - A	Parity	0.84528171	0.55005207	1.29678977	0.44144415	-0.16808532	0.21836159	-0.76975683
proline - A	supplements	3267478.88	6.6167E-05	2.9553E+40	0.97414276	14.9995293	462.763948	0.03241292
proline - A	Weight gain	0.96168255	0.88956104	1.02684181	0.29396624	-0.03907087	0.03722948	-1.04946048
serine - A	serine - A	0.91241425	0.6787372	1.21311995	0.53532942	-0.09166118	0.14786655	-0.6198912
serine - A	energy - A	1.00030401	0.99967807	1.00091063	0.33261121	0.00030397	0.00031373	0.96886769
serine - A	BMI pre-pregnancy	1.06966961	1.02928737	1.1112187	0.00054543	0.06734983	0.01947994	3.4573939
serine - A	ART	0.77353006	0.33996801	1.6364136	0.51916787	-0.25679075	0.39835454	-0.64462864
serine - A	Physical activity A	0.89216417	0.51047044	1.54018223	0.6848231	-0.11410512	0.28112295	-0.40589044
serine - A	Maternal age	1.10375556	1.05448289	1.15706412	2.9872E-05	0.09871851	0.02364831	4.17444228

serine - A	Thyroid disease	0.67925513	0.34337024	1.25003391	0.23737096	-0.38675848	0.32732215	-1.18158361
serine - A	Smoking	2.17215045	1.19366854	3.84436316	0.00902319	0.77571767	0.29707616	2.61117446
serine - A	Parity	0.8354485	0.54398539	1.28073839	0.40956773	-0.17978657	0.2180144	-0.82465457
serine - A	supplements	3171905	6.1205E-05	3.2091E+40	0.97424379	14.9698429	463.660286	0.03228623
serine - A	Weight gain	0.96336638	0.89140282	1.0282799	0.31349155	-0.03732148	0.03702812	-1.00792275
arginine - A	arginine - A	0.97697503	0.74770034	1.28068362	0.86047342	-0.02329418	0.13252534	-0.17577155
arginine - A	energy - A	1.00018778	0.99960163	1.00075936	0.52388761	0.00018776	0.00029459	0.63736425
arginine - A	BMI pre-pregnancy	1.06926964	1.02893519	1.11073067	0.00057616	0.06697584	0.01945504	3.44259506
arginine - A	ART	0.78437034	0.34566687	1.65516576	0.54067708	-0.24287399	0.39698952	-0.61178944
arginine - A	Physical activity A	0.89913037	0.51458906	1.55164752	0.70510293	-0.10632724	0.28096112	-0.37844112
arginine - A	Maternal age	1.10314176	1.0538941	1.15640766	3.3052E-05	0.09816225	0.02364587	4.15134945
arginine - A	Thyroid disease	0.68939789	0.34939009	1.26548231	0.25390561	-0.37193668	0.32599881	-1.14091424
arginine - A	Smoking	2.17380607	1.19362452	3.84911951	0.00902744	0.77647958	0.29738631	2.61101317
arginine - A	Parity	0.82962649	0.54056242	1.27079114	0.39077254	-0.18677969	0.21763686	-0.85821721
arginine - A	supplements	3125149.2	5.9457E-05	3.227E+40	0.9742837	14.9549926	463.919381	0.03223619
arginine - A	Weight gain	0.96355202	0.89165395	1.0283787	0.31512478	-0.0371288	0.03696148	-1.00452677
tyrosine - A	tyrosine - A	0.85388095	0.56445914	1.27289265	0.44316218	-0.1579635	0.20598624	-0.76686433
tyrosine - A	energy - A	1.00033571	0.99972057	1.0009315	0.2759382	0.00033565	0.00030808	1.08948924
tyrosine - A	BMI pre-pregnancy	1.06976139	1.02937304	1.11132811	0.00053801	0.06743563	0.01948399	3.46107937
tyrosine - A	ART	0.77336237	0.34019521	1.63493367	0.51838514	-0.25700756	0.39794513	-0.64583667
tyrosine - A	Physical activity A	0.89452725	0.51213069	1.54341265	0.69144378	-0.11145991	0.28082865	-0.3968965
tyrosine - A	Maternal age	1.10404239	1.05473046	1.15739311	2.8745E-05	0.09897835	0.02366099	4.18318613
tyrosine - A	Thyroid disease	0.67666207	0.341971	1.24554194	0.23294317	-0.39058328	0.32744759	-1.19281159
tyrosine - A	Smoking	2.18120474	1.19894901	3.85962343	0.00863449	0.77987736	0.29696074	2.62619683
tyrosine - A	Parity	0.83569771	0.54426727	1.28080944	0.41009355	-0.17948832	0.21789729	-0.82372901
tyrosine - A	supplements	3184933.16	6.2715E-05	2.7596E+40	0.97421425	14.9739419	463.255683	0.03232328
tyrosine - A	Weight gain	0.96325536	0.89126981	1.02820198	0.31230658	-0.03743673	0.03705162	-1.01039395
EAAs - A	EAAs- A	0.99733884	0.96590669	1.03031802	0.86788143	-0.00266471	0.01601868	-0.16635012
EAAs- A	energy - A	1.00018587	0.99959432	1.00075588	0.52902239	0.00018585	0.00029524	0.62949905
EAAs- A	BMI pre-pregnany	1.0693723	1.02903817	1.11084155	0.00056544	0.06707184	0.01945424	3.44767255
EAAs- A	ART	0.78383883	0.34541224	1.65420794	0.53958958	-0.24355186	0.39703042	-0.61343374
EAAs- A	Physical activity A	0.89836315	0.5142335	1.55016835	0.70278003	-0.1071809	0.28089415	-0.38157041
EAAs- A	Maternal age	1.10316261	1.05387608	1.15647061	3.3407E-05	0.09818115	0.02366434	4.1489069
EAAs- A	Thyroid disease	0.68884696	0.34891779	1.26520358	0.2533143	-0.37273615	0.32629286	-1.14233621
EAAs- A	Smoking	2.17116673	1.19260115	3.84369915	0.00910431	0.77526469	0.29725126	2.60811237

EAAs- A	Parity	0.82959704	0.54054103	1.27074893	0.39068618	-0.18681519	0.21763854	-0.85837364
EAAs- A	supplements	3120790.14	5.9263E-05	3.0876E+40	0.97428735	14.9535968	463.941979	0.03223161
EAAs- A	Weight gain	0.96358589	0.89168442	1.02841357	0.31563221	-0.03709365	0.03696523	-1.00347402
NEAAs- A	NEAAs- A	0.99105521	0.95613073	1.02614403	0.60352059	-0.00898503	0.01730073	-0.5193444
NEAAs- A	energy - A	1.00026976	0.999666	1.00085191	0.37033655	0.00026972	0.00030108	0.89584313
NEAAs- A	BMI pre-pregnancy	1.06964532	1.02925225	1.11120717	0.00054977	0.06732712	0.01948543	3.45525437
NEAAs- A	ART	0.78354033	0.34539209	1.65299934	0.53877493	-0.24393275	0.39685376	-0.61466658
NEAAs- A	Physical activity A	0.89753922	0.51396055	1.54830824	0.7001868	-0.10809846	0.28072539	-0.38506832
NEAAs- A	Maternal age	1.10401902	1.05466225	1.15740239	2.9281E-05	0.09895718	0.0236797	4.17898729
NEAAs- A	Thyroid disease	0.68481067	0.34680929	1.25804016	0.2460543	-0.37861287	0.3263942	-1.1599865
NEAAs- A	Smoking	2.17398861	1.19517729	3.84621765	0.0089021	0.77656355	0.29687521	2.61579118
NEAAs- A	Parity	0.830798	0.54136105	1.27252715	0.39430091	-0.18536859	0.21760872	-0.85184359
NEAAs- A	supplements	3157096.97	6.1555E-05	2.8087E+40	0.97423961	14.9651635	463.440073	0.03229147
NEAAs- A	Weight gain	0.96317336	0.89138401	1.02787438	0.30920484	-0.03752186	0.03689857	-1.01689193
%EAA - A	%EAA - A	1.10421368	0.95440088	1.28634321	0.19215296	0.09913348	0.07600882	1.3042365
%EAA - A	energy - A	1.00016443	0.99977485	1.00054594	0.39862137	0.00016441	0.00019478	0.84408596
%EAA - A	BMI pre-pregnancy	1.06868687	1.02827006	1.1102009	0.00065622	0.06643067	0.01949688	3.40724685
%EAA - A	ART	0.81580662	0.35899858	1.72484722	0.60885393	-0.20357794	0.39783844	-0.51171008
%EAA - A	Physical activity A	0.89380758	0.51123348	1.54335093	0.68979073	-0.11226476	0.28126727	-0.39913908
%EAA - A	Maternal age	1.10203897	1.05299688	1.15507278	3.7457E-05	0.09716207	0.02356798	4.12263051
%EAA - A	Thyroid disease	0.70860632	0.35948328	1.3000708	0.29008667	-0.34445517	0.32559307	-1.05793151
%EAA - A	Smoking	2.17386349	1.19294672	3.85369897	0.00913349	0.776506	0.29785232	2.60701676
%EAA - A	Parity	0.82337874	0.53623131	1.26173998	0.37238564	-0.19433899	0.2178655	-0.8920136
%EAA - A	supplements	3161643.29	6.5918E-05	2.1111E+40	0.97416584	14.9666025	462.160698	0.03238398
%EAA - A	Weight gain	0.96054549	0.88854344	1.02557927	0.27911052	-0.04025393	0.03719228	-1.08231965
BCAAs - A	BCAAs - A	0.97988184	0.90162799	1.06333119	0.62551414	-0.02032329	0.04164179	-0.4880504
BCAAs - A	energy - A	1.00027135	0.99964146	1.00088151	0.38996482	0.00027131	0.0003156	0.85968117
BCAAs - A	BMI pre-pregnancy	1.06951509	1.02916001	1.11102783	0.00055604	0.06720536	0.01946741	3.45219838
BCAAs - A	ART	0.77789312	0.34232749	1.64374484	0.52769946	-0.25116615	0.39771578	-0.63152171
BCAAs - A	Physical activity A	0.89624551	0.51304343	1.54653805	0.69655294	-0.10954089	0.28089005	-0.38997783
BCAAs - A	Maternal age	1.10350756	1.05428553	1.15674989	3.063E-05	0.0984938	0.02362679	4.16873434
BCAAs - A	Thyroid disease	0.68170983	0.34467281	1.25432973	0.24164019	-0.38315118	0.32722876	-1.17089699
BCAAs - A	Smoking	2.17732358	1.19643561	3.85348167	0.00881651	0.7780964	0.29708674	2.61908831
BCAAs - A	Parity	0.83377495	0.54296766	1.27793259	0.40418926	-0.18179176	0.2179332	-0.83416277
BCAAs - A	supplements	3157432.61	6.0898E-05	3.1229E+40	0.97425178	14.9652698	463.662589	0.03227621

BCAAs - A	Weight gain	0.96321641	0.89124729	1.02813038	0.31156611	-0.03747717	0.03703492	-1.01194132
AAAs - A	AAAs - A	0.94890639	0.79964471	1.12143384	0.53907559	-0.05244513	0.08538611	-0.61421148
AAAs - A	energy - A	1.00030544	0.99967059	1.00092032	0.33694505	0.00030539	0.00031805	0.96021891
AAAs - A	BMI pre-pregnancy	1.06953496	1.02917621	1.11105858	0.00055531	0.06722394	0.01947079	3.45255385
AAAs - A	ART	0.77601176	0.34140332	1.6402269	0.52388055	-0.25358761	0.39786243	-0.63737509
AAAs - A	Physical activity A	0.89596443	0.51292883	1.54592435	0.69568179	-0.10985457	0.28084579	-0.39115619
AAAs - A	Maternal age	1.10375798	1.05448893	1.15706037	2.9806E-05	0.09872071	0.02364599	4.17494445
AAAs - A	Thyroid disease	0.67947208	0.34346678	1.25043227	0.23777161	-0.38643914	0.3273313	-1.18057496
AAAs - A	Smoking	2.1787804	1.19751529	3.85543803	0.00873597	0.77876527	0.29698729	2.62221748
AAAs - A	Parity	0.83464713	0.54356849	1.2792165	0.40684187	-0.18074624	0.21790726	-0.82946405
AAAs - A	supplements	3172765.62	6.1889E-05	2.9647E+40	0.97423135	14.9701142	463.444832	0.03230183
AAAs - A	Weight gain	0.9631973	0.89123732	1.02810521	0.31126518	-0.037497	0.03703148	-1.01257087
isoleucine - B	isoleucine - B	1.48510007	1.03111793	2.14528854	0.03374828	0.39548215	0.18627874	2.12306647
isoleucine - B	energy - B	0.99963805	0.99896243	1.00028872	0.28388718	-0.00036201	0.00033782	-1.07162793
isoleucine - B	BMI during pregnancy	1.07135445	1.03052253	1.11340606	0.00045838	0.06892369	0.0196702	3.50396559
isoleucine - B	ART	0.81413465	0.35746751	1.72644673	0.60646329	-0.20562951	0.39918107	-0.51512842
isoleucine - B	Physical activity	1.77835127	0.998001	3.10855472	0.04634885	0.57568668	0.28896997	1.99220243
isoleucine - B	Maternal age	1.10301546	1.05347044	1.15658199	3.7528E-05	0.09804776	0.02378533	4.12219426
isoleucine - B	Thyroid disease	0.70228115	0.35554448	1.2919278	0.27951456	-0.35342146	0.32681527	-1.08141049
isoleucine - B	Smoking	2.09405984	1.14655293	3.72032371	0.0134388	0.73910469	0.29899975	2.4719241
isoleucine - B	Parity	0.83541271	0.54335533	1.28203872	0.41064403	-0.17982942	0.21856828	-0.82276081
isoleucine - B	supplements	3020943.81	6.4945E-05	1.9413E+40	0.97421313	14.9210799	461.600257	0.03232468
isoleucine - B	Weight gain	0.9409673	0.86922311	1.01054486	0.12085523	-0.06084689	0.03922585	-1.55119387
leucine - B	leucine - B	1.2222019	0.97702764	1.53107687	0.07870238	0.20065407	0.11412048	1.75826527
leucine - B	energy - B	0.99971499	0.99903468	1.00037143	0.40246228	-0.00028505	0.00034046	-0.83723181
leucine - B	BMI during pregnancy	1.07145124	1.03062214	1.11351058	0.00045047	0.06901403	0.01966997	3.50859835
leucine - B	ART	0.80873773	0.3554915	1.71263865	0.59428246	-0.2122806	0.39854386	-0.5326405
leucine - B	Physical activity	1.78069943	0.99727188	3.12021913	0.04671192	0.57700622	0.29011282	1.98890289
leucine - B	Maternal age	1.10420199	1.05468043	1.157758	3.0027E-05	0.0991229	0.02375192	4.17325837
leucine - B	Thyroid disease	0.70475768	0.35675066	1.29664553	0.28442635	-0.34990125	0.32687953	-1.07042878
leucine - B	Smoking	2.10681638	1.15556688	3.73723585	0.01244648	0.74517798	0.29816348	2.49922618
leucine - B	Parity	0.83458775	0.54314395	1.28001257	0.40742706	-0.18081739	0.21826515	-0.82842994
leucine - B	supplements	3029514.96	6.1662E-05	2.2089E+40	0.97426484	14.9239131	462.615903	0.03225984

leucine - B	Weight gain	0.94072733	0.86905389	1.01016817	0.11887298	-0.06110195	0.03917993	-1.5595216
valine - B	valine - B	1.31396003	0.9717808	1.77931333	0.07538047	0.2730455	0.1535566	1.77814239
valine - B	energy - B	0.99971566	0.99903789	1.00036835	0.40125724	-0.00028438	0.0003388	-0.83937797
valine - B	BMI during pregnancy	1.07202587	1.03120121	1.11409166	0.00040377	0.0695502	0.01966022	3.53761041
valine - B	ART	0.81177495	0.3566612	1.71990768	0.60103618	-0.20853213	0.39879086	-0.52291101
valine - B	Physical activity	1.78024822	0.99730411	3.11826552	0.04668148	0.57675281	0.28994519	1.98917871
valine - B	Maternal age	1.10383845	1.05432303	1.15738578	3.2018E-05	0.0987936	0.02375635	4.158619
valine - B	Thyroid disease	0.70273771	0.35580065	1.29258337	0.28031888	-0.35277156	0.32676036	-1.07960329
valine - B	Smoking	2.09755953	1.1503248	3.7208917	0.01298833	0.74077454	0.29820771	2.48408916
valine - B	Parity	0.82959992	0.5396968	1.27270701	0.39241009	-0.18681172	0.21842814	-0.85525483
valine - B	supplements	3034159.73	6.3138E-05	2.1183E+40	0.97423945	14.9254451	462.207241	0.03229167
valine - B	Weight gain	0.94080728	0.86912003	1.01024816	0.11940721	-0.06101697	0.03918209	-1.55726658
lysine - B	lysine - B	1.21772931	0.96580359	1.53541385	0.09438754	0.19698791	0.11776685	1.67269404
lysine - B	energy - B	0.99979175	0.99915732	1.0004029	0.51144567	-0.00020827	0.0003172	-0.6565883
lysine - B	BMI during pregnancy	1.07135973	1.03056027	1.11343959	0.00045818	0.06892862	0.01967094	3.50408418
lysine - B	ART	0.80635432	0.35416176	1.70884476	0.5895396	-0.21523203	0.39894476	-0.53950333
lysine - B	Physical activity	1.74978487	0.982619	3.05575299	0.05251869	0.55949285	0.28856831	1.93885754
lysine - B	Maternal age	1.10443171	1.05492536	1.15797404	2.8635E-05	0.09933091	0.02374031	4.1840604
lysine - B	Thyroid disease	0.69876702	0.3537424	1.28535123	0.272742	-0.3584379	0.32681231	-1.09676988
lysine - B	Smoking	2.09422687	1.14966756	3.71131255	0.01302844	0.73918445	0.29769923	2.48299083
lysine - B	Parity	0.8353496	0.54377273	1.28091305	0.40955062	-0.17990496	0.21814999	-0.8246847
lysine - B	supplements	3038008.01	6.341E-05	2.1421E+40	0.97423437	14.9267126	462.155409	0.03229804
lysine - B	Weight gain	0.94084864	0.8692947	1.01015009	0.11892956	-0.060973	0.03910325	-1.55928237
methionine - B	methionine - B	1.79315711	0.805972	3.98558272	0.15060842	0.58397782	0.40627773	1.43738574
methionine - B	energy - B	0.99982446	0.99917848	1.00044919	0.58741676	-0.00017556	0.00032356	-0.54258328
methionine - B	BMI during pregnancy	1.07183173	1.03103903	1.11387243	0.00041519	0.06936908	0.01965	3.53023247
methionine - B	ART	0.79955568	0.35145783	1.69307818	0.57458466	-0.2236991	0.39852878	-0.5613123
methionine - B	Physical activity	1.7534191	0.98217855	3.07136941	0.05279524	0.56156765	0.28997727	1.93659198
methionine - B	Maternal age	1.10545647	1.05598999	1.15896683	2.3367E-05	0.10025834	0.02370161	4.23002177
methionine - B	Thyroid disease	0.70257224	0.35565023	1.29252655	0.28013991	-0.35300705	0.32685684	-1.0800051
methionine - B	Smoking	2.09716458	1.15156203	3.7157562	0.01282273	0.74058623	0.29758492	2.48865512
methionine - B	Parity	0.83430729	0.54320019	1.27903499	0.40608102	-0.1811535	0.21804446	-0.83080991

methionine - B	supplements	3091136.06	6.4419E-05	1.7719E+40	0.97420298	14.9440492	462.128878	0.03233741
methionine - B	Weight gain	0.94044373	0.86889005	1.00980489	0.11658356	-0.06140346	0.03912852	-1.56927637
phenylalanine - B	phenylalanine - B	1.60093316	1.0476129	2.45736345	0.0299539	0.47058669	0.21679034	2.17069947
phenylalanine - B	energy - B	0.99958752	0.99888435	1.00026402	0.24044832	-0.00041257	0.00035146	-1.17386695
phenylalanine - B	BMI during pregnancy	1.0718749	1.0310231	1.11392938	0.00041641	0.06940936	0.01966574	3.52945634
phenylalanine - B	ART	0.81078879	0.35624356	1.71773988	0.59889222	-0.20974769	0.39876424	-0.52599422
phenylalanine - B	Physical activity	1.79391628	1.00548777	3.14074022	0.04365967	0.58440109	0.28968843	2.01734358
phenylalanine - B	Maternal age	1.10253969	1.05305881	1.1560457	4.0035E-05	0.09761633	0.02376669	4.10727503
phenylalanine - B	Thyroid disease	0.70695539	0.3578302	1.3009602	0.28884568	-0.34678771	0.32695548	-1.06065728
phenylalanine - B	Smoking	2.12718851	1.16420926	3.78203786	0.01167102	0.75480116	0.29929406	2.52193833
phenylalanine - B	Parity	0.83568719	0.54347922	1.28258804	0.41160831	-0.17950091	0.21861917	-0.82106663
phenylalanine - B	supplements	3004051.45	6.373E-05	2.113E+40	0.97423744	14.9154724	461.862422	0.03229419
phenylalanine - B	Weight gain	0.94036604	0.86858327	1.01008237	0.1176468	-0.06148608	0.03929507	-1.56472757
threonine - B	threonine - B	1.56154124	1.00381606	2.43613492	0.04808292	0.44567331	0.22547074	1.97663481
threonine - B	energy - B	0.99966832	0.99898964	1.00032156	0.32814014	-0.00033173	0.00033924	-0.97786694
threonine - B	BMI during pregnancy	1.07188939	1.03106037	1.11396184	0.0004153	0.06942287	0.01966562	3.53016511
threonine - B	ART	0.81505322	0.35785821	1.7284339	0.60845208	-0.20450187	0.39919609	-0.51228426
threonine - B	Physical activity	1.78150435	0.99878399	3.11770587	0.046093	0.57745815	0.28951936	1.99454068
threonine - B	Maternal age	1.10357223	1.05400958	1.15716074	3.4144E-05	0.0985524	0.0237825	4.14390429
threonine - B	Thyroid disease	0.70088043	0.35496633	1.28878094	0.2765013	-0.35541797	0.32660713	-1.08821252
threonine - B	Smoking	2.07503407	1.13668481	3.68390695	0.01453129	0.72997757	0.2986993	2.44385434
threonine - B	Parity	0.83249164	0.54153859	1.27734112	0.40141332	-0.1833321	0.21848666	-0.83909978
threonine - B	supplements	3012383.01	6.3727E-05	2.1232E+40	0.9742355	14.918242	461.91335	0.03229662
threonine - B	Weight gain	0.94087217	0.86917451	1.01040349	0.12003271	-0.06094799	0.03920402	-1.5546363
tryptophane - B	tryptophane - B	2.91563074	0.67006416	12.7118053	0.15206691	1.07008617	0.74712663	1.43226881
tryptophane - B	energy - B	0.99980543	0.99913641	1.00044935	0.56043262	-0.00019459	0.00033424	-0.58219904
tryptophane - B	BMI during pregnancy	1.07251812	1.03170427	1.11459118	0.00036723	0.07000927	0.01965129	3.56257967
tryptophane - B	ART	0.8017148	0.35237865	1.69766923	0.57923006	-0.22100235	0.39855451	-0.55450972
tryptophane - B	Physical activity	1.76049889	0.98543636	3.08539825	0.05137743	0.56559723	0.29030089	1.9483138
tryptophane - B	Maternal age	1.10511364	1.05566329	1.15860877	2.4766E-05	0.09994817	0.02370169	4.2169211
tryptophane - B	Thyroid disease	0.69943986	0.35404331	1.28669106	0.27410415	-0.35747546	0.32686162	-1.09365994
tryptophane - B	Smoking	2.08178252	1.1428668	3.68835646	0.01375771	0.73322451	0.29763189	2.46352804

tryptophane - B	Parity	0.83190565	0.54155862	1.27551446	0.39880013	-0.18403625	0.21811287	-0.84376609
tryptophane - B	supplements	3046162.85	6.1667E-05	2.2795E+40	0.97426116	14.9293933	462.719661	0.03226445
tryptophane - B	Weight gain	0.94107099	0.86947735	1.01031474	0.12030315	-0.0607367	0.03909663	-1.55350241
histidine - B	histidine - B	1.77620564	0.89352608	3.54141378	0.10077994	0.57447942	0.35006091	1.64108418
histidine - B	energy - B	0.99975587	0.99908586	1.00040112	0.4660084	-0.00024416	0.00033493	-0.72898898
histidine - B	BMI during pregnancy	1.07197281	1.03115625	1.11404893	0.00040831	0.06950069	0.01966266	3.53465378
histidine - B	ART	0.79848208	0.35096777	1.69048573	0.57225733	-0.22504275	0.39849608	-0.56473015
histidine - B	Physical activity	1.78614174	0.99835681	3.13682358	0.04636702	0.58005784	0.29118831	1.99203684
histidine - B	Maternal age	1.10566317	1.05619228	1.15918715	2.2553E-05	0.10044531	0.02370115	4.23799361
histidine - B	Thyroid disease	0.70696153	0.35776141	1.30119621	0.28899741	-0.34677903	0.32705019	-1.06032359
histidine - B	Smoking	2.08485918	1.14378202	3.69624777	0.01367502	0.73470132	0.29797004	2.46568857
histidine - B	Parity	0.83289997	0.54218395	1.27713064	0.40193072	-0.18284173	0.21814188	-0.83817804
histidine - B	supplements	3021485.13	6.1536E-05	2.3833E+40	0.97426995	14.921259	462.625629	0.03225342
histidine - B	Weight gain	0.93989756	0.8684737	1.00909974	0.11264384	-0.06198438	0.03907184	-1.58642098
alanine - B	alanine - B	1.38749985	0.93550716	2.05704737	0.1024315	0.32750346	0.20053136	1.63317831
alanine - B	energy - B	0.99973844	0.99905631	1.0004004	0.4448336	-0.00026159	0.00034237	-0.76405646
alanine - B	BMI during pregnancy	1.07220925	1.03140321	1.11427451	0.00038882	0.06972124	0.01965332	3.547555
alanine - B	ART	0.79617965	0.34981187	1.68688099	0.5676287	-0.22793043	0.39879538	-0.57154731
alanine - B	Physical activity	1.75153161	0.98277909	3.06210014	0.05249376	0.56049061	0.28905241	1.93906226
alanine - B	Maternal age	1.10453069	1.05501238	1.15808161	2.8221E-05	0.09942053	0.02374298	4.18736452
alanine - B	Thyroid disease	0.69316259	0.35092647	1.27481676	0.26203195	-0.36649069	0.32675662	-1.12160141
alanine - B	Smoking	2.08284603	1.14258984	3.69306552	0.01381387	0.73373524	0.2980159	2.46206745
alanine - B	Parity	0.83731396	0.54505448	1.28402183	0.41573017	-0.17755618	0.21816786	-0.81385125
alanine - B	supplements	3062260.75	6.4277E-05	1.8602E+40	0.97421283	14.934664	462.015195	0.03232505
alanine - B	Weight gain	0.94065991	0.86911353	1.01017249	0.11814323	-0.06117362	0.03914824	-1.56261477
aspartic acid - B	aspartic acid - B	1.32568243	1.05487548	1.66900665	0.01579012	0.28192737	0.11680149	2.41373081
aspartic acid - B	energy - B	0.99953406	0.99883729	1.00020637	0.18154939	-0.00046605	0.00034884	-1.33599965
aspartic acid - B	BMI during pregnancy	1.07209384	1.03125208	1.11416567	0.00039979	0.06961359	0.01966363	3.54022056
aspartic acid - B	ART	0.80350338	0.35212019	1.70674628	0.58453237	-0.21877388	0.40011583	-0.54677638
aspartic acid - B	Physical activity	1.73829338	0.9775003	3.02974863	0.05464122	0.55290382	0.28771326	1.92171822
aspartic acid - B	Maternal age	1.10093895	1.05148214	1.1544019	5.2784E-05	0.09616341	0.02378548	4.04294618
aspartic acid - B	Thyroid disease	0.69579271	0.35202905	1.28089268	0.26759904	-0.36270349	0.32716999	-1.10860866

aspartic acid - B	Smoking	2.12441058	1.16150146	3.78034453	0.01195065	0.75349439	0.29976724	2.51359816
aspartic acid - B	Parity	0.85548239	0.55631132	1.31357407	0.47551494	-0.15608977	0.21875568	-0.71353471
aspartic acid - B	supplements	3014812.77	7.1749E-05	1.6212E+40	0.97411164	14.9190483	459.727464	0.03245194
aspartic acid - B	Weight gain	0.94032547	0.86861806	1.01005758	0.11717199	-0.06152922	0.03927176	-1.56675489
glutamic acid - B	glutamic acid - B	1.10076845	0.98553345	1.23064478	0.08928005	0.09600853	0.05650194	1.69920775
glutamic acid - B	energy - B	0.99964605	0.99889099	1.00037353	0.34853204	-0.00035402	0.00037764	-0.93744045
glutamic acid - B	BMI during pregnancy	1.07267896	1.03179746	1.11479478	0.00036239	0.07015922	0.01967415	3.5660614
glutamic acid - B	ART	0.79562613	0.35006954	1.68294476	0.56567342	-0.22862589	0.39800127	-0.57443508
glutamic acid - B	Physical activity	1.81117609	1.01014517	3.18884997	0.04220639	0.59397641	0.29238614	2.03147938
glutamic acid - B	Maternal age	1.10542007	1.05585799	1.15903491	2.4398E-05	0.10022542	0.02374842	4.22029813
glutamic acid - B	Thyroid disease	0.70074389	0.35493102	1.28830524	0.27613566	-0.35561281	0.32653748	-1.08904134
glutamic acid - B	Smoking	2.07847808	1.13940916	3.68749863	0.0141933	0.73163593	0.29834274	2.4523336
glutamic acid - B	Parity	0.82427136	0.53610634	1.26476785	0.3765216	-0.19325548	0.21853483	-0.88432351
glutamic acid - B	supplements	2981327.16	5.5802E-05	3.058E+40	0.97438078	14.9078791	464.211016	0.03211444
glutamic acid - B	Weight gain	0.94202496	0.87028653	1.01161852	0.12739691	-0.0597235	0.03917714	-1.52444771
cysteine - B	cysteine - B	5.75976209	1.42296453	23.4649087	0.01384876	1.75089617	0.71141017	2.46116268
cysteine - B	energy - B	0.99971741	0.99913	1.00028573	0.33694818	-0.00028263	0.00029434	-0.96021269
cysteine - B	BMI during pregnancy	1.07358426	1.03255716	1.11582447	0.00031816	0.07100283	0.01972277	3.60004284
cysteine - B	ART	0.78937132	0.34643539	1.67511649	0.5538125	-0.23651845	0.39948603	-0.59205688
cysteine - B	Physical activity	1.73800941	0.97684763	3.03115148	0.05495062	0.55274044	0.28799567	1.91926649
cysteine - B	Maternal age	1.10509141	1.05544621	1.15878431	2.6689E-05	0.09992805	0.02379226	4.20002349
cysteine - B	Thyroid disease	0.70654669	0.35762748	1.30018133	0.2880335	-0.34736599	0.32694947	-1.06244547
cysteine - B	Smoking	2.07300781	1.13070397	3.69440972	0.01535371	0.7290006	0.30075192	2.42392667
cysteine - B	Parity	0.8492898	0.55203386	1.30463712	0.45567931	-0.16335481	0.21898006	-0.74598031
cysteine - B	supplements	3089792.09	7.2959E-05	1.2377E+40	0.97407262	14.9436144	459.791177	0.03250087
cysteine - B	Weight gain	0.94161449	0.86953675	1.01151394	0.126771	-0.06015934	0.03939812	-1.52695982
glycine - B	glycine - B	1.55171953	0.96992325	2.46803673	0.06382937	0.43936369	0.23706207	1.85336981
glycine - B	energy - B	0.99975351	0.9991262	1.0003662	0.43492501	-0.00024652	0.00031573	-0.78079172
glycine - B	BMI during pregnancy	1.07230298	1.03134871	1.11449025	0.00039962	0.06980865	0.01971811	3.54033261
glycine - B	ART	0.7498702	0.32791621	1.59502533	0.47291838	-0.28785516	0.4010584	-0.71773876
glycine - B	Physical activity	1.68200258	0.94841379	2.92152636	0.06918615	0.51998509	0.28614597	1.81720223
glycine - B	Maternal age	1.1044337	1.05495169	1.15795047	2.8375E-05	0.09933271	0.02372901	4.18613021

glycine - B	Thyroid disease	0.67129268	0.33813801	1.23933871	0.22580556	-0.39855005	0.32904452	-1.21123445
glycine - B	Smoking	2.06871894	1.13329899	3.67218737	0.01493102	0.72692955	0.29865046	2.43404797
glycine - B	Parity	0.85429608	0.55573434	1.31145962	0.47130004	-0.15747744	0.21860772	-0.72036541
glycine - B	supplements	3069266.19	6.4362E-05	1.6449E+40	0.97420801	14.9369491	461.999521	0.03233109
glycine - B	Weight gain	0.94226025	0.8705435	1.01206376	0.12924255	-0.05947377	0.03920239	-1.5170956
proline - B	proline - B	1.20945235	0.93662518	1.56194187	0.14248455	0.19016766	0.12966554	1.4666013
proline - B	energy - B	0.99976664	0.99907044	1.00044092	0.50366168	-0.00023339	0.00034899	-0.66873947
proline - B	BMI during pregnancy	1.07178644	1.03092037	1.11389034	0.00042842	0.06932683	0.01968433	3.52192955
proline - B	ART	0.79958649	0.35184439	1.69131902	0.574123	-0.22366057	0.39797979	-0.56198976
proline - B	Physical activity	1.75761761	0.98495525	3.07627336	0.05154479	0.56395926	0.28966798	1.94691612
proline - B	Maternal age	1.1053221	1.05578481	1.15890679	2.4607E-05	0.10013678	0.02373823	4.2183769
proline - B	Thyroid disease	0.6966274	0.35264221	1.28121685	0.26862404	-0.36150459	0.32678771	-1.1062368
proline - B	Smoking	2.10420248	1.15581403	3.72766829	0.01238662	0.74393653	0.29746344	2.50093429
proline - B	Parity	0.83017348	0.54024428	1.27318952	0.39381388	-0.18612059	0.21826662	-0.85272129
proline - B	supplements	3028540.48	5.6321E-05	2.6562E+40	0.97435792	14.9235914	464.285773	0.03214312
proline - B	Weight gain	0.94246985	0.87067552	1.011847	0.13005496	-0.05925135	0.03913861	-1.51388518
serine - B	serine - B	1.25373571	0.91639577	1.71446773	0.15411868	0.22612767	0.15867124	1.42513327
serine - B	energy - B	0.99980452	0.99913474	1.00045162	0.55979401	-0.0001955	0.00033525	-0.5831475
serine - B	BMI during pregnancy	1.07220119	1.03138137	1.11427123	0.00039043	0.06971372	0.01965723	3.54646677
serine - B	ART	0.80431338	0.35354593	1.70282033	0.58473376	-0.21776632	0.39848667	-0.54648331
serine - B	Physical activity	1.75090422	0.98124629	3.06459443	0.05314433	0.56013235	0.28966176	1.93374631
serine - B	Maternal age	1.10419183	1.05469846	1.15772571	2.9816E-05	0.09911369	0.02374057	4.17486615
serine - B	Thyroid disease	0.69886776	0.35384717	1.28523149	0.2727891	-0.35829373	0.32671295	-1.09666216
serine - B	Smoking	2.11575605	1.16210174	3.74845537	0.01176655	0.74941222	0.29749569	2.51906919
serine - B	Parity	0.82860143	0.53914625	1.27091765	0.38915142	-0.18801602	0.21832948	-0.86115728
serine - B	supplements	3057564.63	6.135E-05	2.1835E+40	0.97426292	14.9331293	462.86712	0.03226224
serine - B	Weight gain	0.94126905	0.86953605	1.01076718	0.12248923	-0.06052626	0.03919056	-1.54440921
arginine - B	arginine - B	1.53362993	1.11305462	2.10450943	0.00810689	0.42763743	0.16151979	2.64758542
arginine - B	energy - B	0.99957973	0.99893623	1.00020261	0.19255188	-0.00042035	0.00032259	-1.30306703
arginine - B	BMI during pregnancy	1.07407173	1.03306995	1.11630071	0.00028737	0.07145678	0.01970444	3.62643065
arginine - B	ART	0.77666704	0.34000876	1.65063179	0.52802991	-0.25274354	0.40053413	-0.63101624
arginine - B	Physical activity	1.72853675	0.97120401	3.01456994	0.05746329	0.54727524	0.28807445	1.89977015

arginine - B	Maternal age	1.10124509	1.05196326	1.1545127	4.6881E-05	0.09644144	0.02369187	4.07065582
arginine - B	Thyroid disease	0.69640491	0.35207713	1.28302444	0.2693371	-0.36182402	0.32756396	-1.10459043
arginine - B	Smoking	2.13353526	1.16497167	3.80119435	0.01165062	0.75778035	0.30040207	2.52255366
arginine - B	Parity	0.85567465	0.55617804	1.3146215	0.47667889	-0.15586506	0.21901795	-0.71165427
arginine - B	supplements	3028240.13	7.5721E-05	1.3457E+40	0.974051	14.9234922	458.78929	0.03252799
arginine - B	Weight gain	0.93890988	0.86710757	1.00927051	0.11041212	-0.06303578	0.03948761	-1.59634351
tyrosine - B	tyrosine - B	1.38778638	0.87478974	2.2026322	0.16207731	0.32770994	0.23439344	1.39811909
tyrosine - B	energy - B	0.99981473	0.99914862	1.0004572	0.57811792	-0.00018528	0.00033316	-0.55613595
tyrosine - B	BMI during pregnancy	1.07198211	1.03114433	1.1140554	0.00040858	0.06950938	0.01966609	3.53447888
tyrosine - B	ART	0.80263757	0.35277296	1.69972785	0.58122731	-0.21985201	0.39857658	-0.55159289
tyrosine - B	Physical activity	1.75315964	0.98185349	3.07098192	0.05290341	0.56141967	0.29003316	1.93570856
tyrosine - B	Maternal age	1.10456079	1.05506197	1.15809895	2.7903E-05	0.09944778	0.02373489	4.18994053
tyrosine - B	Thyroid disease	0.70089758	0.35490042	1.28895491	0.27665609	-0.35539351	0.32668992	-1.08786188
tyrosine - B	Smoking	2.09620261	1.15123593	3.713402	0.01285136	0.74012743	0.29749537	2.487862
tyrosine - B	Parity	0.82973143	0.53998528	1.27245908	0.39240964	-0.1866532	0.21824259	-0.85525563
tyrosine - B	supplements	3054434.78	6.2196E-05	2.1759E+40	0.97424995	14.9321051	462.602202	0.0322785
tyrosine - B	Weight gain	0.94083685	0.86916518	1.01039509	0.11970283	-0.06098553	0.03919323	-1.55602211
EAAs- B	EAAs- B	1.04937768	1.00777292	1.09301181	0.0196791	0.0481973	0.02066416	2.33241062
EAAs- B	energy - B	0.99960715	0.99894632	1.00024676	0.23560441	-0.00039293	0.00033129	-1.18604531
EAAs- B	BMI during pregnancy	1.07149576	1.0306833	1.11351504	0.0004425	0.06905558	0.01965521	3.51334762
EAAs- B	ART	0.80682821	0.35379621	1.71340207	0.59143193	-0.21464451	0.39988758	-0.53676213
EAAs- B	Physical activity	1.73180295	0.97393639	3.01819676	0.0562626	0.54916303	0.28767112	1.90899607
EAAs- B	Maternal age	1.10141731	1.05188401	1.1549555	4.9721E-05	0.09659781	0.02381054	4.0569357
EAAs- B	Thyroid disease	0.6995621	0.35405585	1.28741619	0.27453717	-0.35730071	0.32699676	-1.09267353
EAAs- B	Smoking	2.11750948	1.1584492	3.76578198	0.01223256	0.75024063	0.29945381	2.50536343
EAAs- B	Parity	0.85431189	0.55552343	1.31180888	0.47168946	-0.15745894	0.21877412	-0.71973291
EAAs- B	supplements	3055336.56	7.1987E-05	1.4125E+40	0.9740964	14.9324003	459.86801	0.03247106
EAAs- B	Weight gain	0.93955089	0.86786189	1.00936214	0.11270358	-0.0623533	0.03931091	-1.58615753
NEAAs- B	NEAAs- B	1.05642354	1.00657379	1.10906915	0.02617391	0.05488918	0.02468459	2.22362163
NEAAs- B	energy - B	0.99957058	0.99887401	1.00024817	0.21993125	-0.00042951	0.00035013	-1.22671094
NEAAs- B	BMI during pregnancy	1.07157193	1.03073414	1.11360264	0.00043873	0.06912666	0.01966274	3.51561618
NEAAs- B	ART	0.79252308	0.34760825	1.6826508	0.56079671	-0.23253366	0.39977692	-0.58165853

NEAAs- B	Physical activity	1.71989318	0.96806713	2.99433796	0.0590048	0.54226218	0.28719117	1.88815759
NEAAs- B	Maternal age	1.10163241	1.05208327	1.15518906	4.8123E-05	0.09679309	0.02381392	4.0645591
NEAAs- B	Thyroid disease	0.69039677	0.34925835	1.27081826	0.25747294	-0.37048882	0.32717589	-1.13238422
NEAAs- B	Smoking	2.1174027	1.15954882	3.76228059	0.01210161	0.7501902	0.29897973	2.5091674
NEAAs- B	Parity	0.85586438	0.55657888	1.31413072	0.47674934	-0.15564335	0.21874137	-0.71154054
NEAAs- B	supplements	3054793.74	6.7033E-05	1.5918E+40	0.9741702	14.9322226	461.176928	0.03237851
NEAAs- B	Weight gain	0.94034826	0.86862644	1.01032598	0.11773305	-0.06150498	0.03931638	-1.56436
%EAA - B	%EAA - B	1.10214768	0.95041807	1.28986883	0.21294479	0.09726072	0.07808914	1.24550879
%EAA - B	energy - B	1.00013822	0.99967287	1.00059263	0.55478624	0.00013821	0.00023401	0.59060332
%EAA - B	BMI during pregnancy	1.07186217	1.03102993	1.11398643	0.00042192	0.06939748	0.01968177	3.5259769
%EAA - B	ART	0.80200731	0.35227616	1.69944879	0.58017836	-0.22063756	0.39889334	-0.55312421
%EAA - B	Physical activity	1.67575046	0.94615237	2.90754031	0.0706037	0.5162611	0.2855398	1.80801798
%EAA - B	Maternal age	1.1069375	1.05748986	1.16045065	1.763E-05	0.1015972	0.02366593	4.29297213
%EAA - B	Thyroid disease	0.70494129	0.3565307	1.29828582	0.28549201	-0.34964076	0.32735962	-1.06806319
%EAA - B	Smoking	2.09902043	1.15579634	3.70984316	0.01231986	0.74147077	0.29625087	2.50284762
%EAA - B	Parity	0.83267619	0.5422921	1.27619362	0.40072652	-0.18311044	0.21790446	-0.84032442
%EAA - B	supplements	3180197.55	7.3875E-05	1.2207E+40	0.97403855	14.9724539	460.073778	0.03254359
%EAA - B	Weight gain	0.94004679	0.86844735	1.00917737	0.11391831	-0.06182563	0.03910975	-1.5808239
BCAAS - B	BCAAS - B	1.09549325	0.99559792	1.20619929	0.06136134	0.09120472	0.04874956	1.87088275
BCAAS - B	energy - B	0.99968926	0.99900861	1.00034521	0.36125345	-0.00031079	0.00034041	-0.91297925
BCAAS - B	BMI during pregnancy	1.07158175	1.03075537	1.11363814	0.00043898	0.06913583	0.01966618	3.5154679
BCAAS - B	ART	0.81173589	0.35666759	1.71981638	0.600933	-0.20858025	0.39876981	-0.52305929
BCAAS - B	Physical activity	1.7833417	0.99931614	3.12282632	0.04591833	0.57848896	0.28980333	1.99614326
BCAAS - B	Maternal age	1.10375389	1.05422829	1.15730963	3.2625E-05	0.098717	0.02376245	4.15432778
BCAAS - B	Thyroid disease	0.70390286	0.35635483	1.29492484	0.28267811	-0.35111492	0.32682447	-1.07432262
BCAAS - B	Smoking	2.10064705	1.15157648	3.72789675	0.01286959	0.74224542	0.29840719	2.48735772
BCAAS - B	Parity	0.83300698	0.54197216	1.27787696	0.4027954	-0.18271326	0.21838954	-0.83663921
BCAAS - B	supplements	3026021.67	6.2821E-05	2.1298E+40	0.97424654	14.9227593	462.251394	0.03228278
BCAAS - B	Weight gain	0.94080642	0.86911325	1.01026943	0.11947035	-0.06101788	0.03918937	-1.55700058
AAAs - B	AAAs - B	1.20232953	0.97816379	1.47959225	0.07995225	0.18426095	0.10523405	1.75096317
AAAs - B	energy - B	0.99970768	0.99901946	1.00037131	0.39579301	-0.00029236	0.0003443	-0.84915867
AAA - B	BMI during pregnancy	1.07140953	1.03054936	1.1134723	0.00045632	0.0689751	0.01967813	3.50516621

AAAs - B	ART	0.81039154	0.3561373	1.71664397	0.59795789	-0.21023777	0.39867635	-0.52733945
AAAs - B	Physical activity	1.78003676	0.99730984	3.11758567	0.04668044	0.57663401	0.28988409	1.98918818
AAAs - B	Maternal age	1.10368758	1.05424563	1.1571626	3.2073E-05	0.09865692	0.02372572	4.1582258
AAAs - B	Thyroid disease	0.70445287	0.35669671	1.29564611	0.28359846	-0.35033384	0.32672145	-1.07227071
AAAs - B	Smoking	2.07933284	1.1393911	3.69083309	0.01421481	0.73204709	0.29857674	2.45178874
AAAs - B	Parity	0.82837921	0.53890072	1.27081446	0.38868215	-0.18828424	0.21842473	-0.86200975
AAAs - B	supplements	3034443.79	6.1837E-05	2.0876E+40	0.97425999	14.9255387	462.579112	0.03226592
AAAs - B	Weight gain	0.94079822	0.86916665	1.01002784	0.11876861	-0.06102659	0.03912054	-1.55996308

A refers to Period A (pre-pregnancy) and B refers to Period B (during pregnancy); Thyroid disease includes hypothyroidism, Hashimoto's thyroiditis, hyperthyroidism; BMI during pregnancy measured at mid-gestation 21-23 weeks of gestation
